# *Pseudomonas aeruginosa* heme metabolites biliverdin IXβ and IXδ are integral to lifestyle adaptations associated with chronic infection

**DOI:** 10.1128/mbio.02763-23

**Published:** 2024-02-06

**Authors:** Saba Shahzad, Samuel A. Krug, Susana Mouriño, Weiliang Huang, Maureen A. Kane, Angela Wilks

**Affiliations:** 1 Department of Pharmaceutical Sciences, School of Pharmacy, University of Maryland, Baltimore, Maryland, USA; California Institute of Technology, Pasadena, California, USA

**Keywords:** *Pseudomonas*, heme, biliverdin, proteomics, metabolomics, chemotaxis

## Abstract

*Pseudomonas aeruginosa* is a versatile opportunistic pathogen requiring iron for its survival and virulence within the host. The ability to switch to heme as an iron source and away from siderophore uptake provides an advantage in chronic infection. We have recently shown the extracellular heme metabolites biliverdin IXβ (BVIXβ) and BVIXδ positively regulate the heme-dependent cell surface signaling cascade. We further investigated the role of BVIXβ and BVIXδ in cell signaling utilizing allelic strains lacking a functional heme oxygenase (*hemO*in) or one reengineered to produce BVIXα (*hemO*α). Compared to PAO1, both strains show a heme-dependent growth defect, decreased swarming and twitching, and less robust biofilm formation. Interestingly, the motility and biofilm defects were partially rescued on addition of exogenous BVIXβ and BVIXδ. Utilizing liquid chromatography-tandem mass spectrometry, we performed a comparative proteomics and metabolomics analysis of PAO1 versus the allelic strains in shaking and static conditions. In shaking conditions, the *hemO* allelic strains showed a significant increase in proteins involved in quorum sensing, phenazine production, and chemotaxis. Metabolite profiling further revealed increased levels of *Pseudomonas* quinolone signal and phenazine metabolites. In static conditions, we observed a significant repression of chemosensory pathways and type IV pili biogenesis proteins as well as several phosphodiesterases associated with biofilm dispersal. We propose BVIX metabolites function as signaling and chemotactic molecules integrating heme utilization as an iron source into the adaptation of *P. aeruginosa* from a planktonic to sessile lifestyle.

**IMPORTANCE:**

The opportunistic pathogen *Pseudomonas aeruginosa* causes long-term chronic infection in the airways of cystic fibrosis patients. The ability to scavenge iron and to establish chronic infection within this environment coincides with a switch to utilize heme as the primary iron source. Herein, we show the heme metabolites biliverdin beta and delta are themselves important signaling molecules integrating the switch in iron acquisition systems with cooperative behaviors such as motility and biofilm formation that are essential for long-term chronic infection. These significant findings will enhance the development of viable multi-targeted therapeutics effective against both heme utilization and cooperative behaviors essential for survival and persistence within the host.

## INTRODUCTION

*Pseudomonas aeruginosa* is a versatile opportunistic pathogen capable of causing a wide variety of acute and chronic infections. In cystic fibrosis (CF) patients, it is a major cause of morbidity and mortality leading to lifelong chronic infections that are recalcitrant to antibiotic therapy (1). *P. aeruginosa* chronic infection is characterized by the formation of biofilms that require coordinated response of the quorum-sensing (QS) network of diffusible signaling molecules that regulate several virulence factors including proteases and exotoxins, as well as cell-associated virulence factors such as flagella, type IV pili (TFP), and adhesins (2, 3). The hierarchy of the *P. aeruginosa* QS systems is governed by the *N*-(3-oxododecanoyl)-L-homoserine lactone and the *N*-butanoyl-L-homoserine lactone responsive LasR and RhlR regulators, respectively (4, 5). A third chemically distinct signaling molecule, 2-heptyl-3-hydroxy-4(1*H*)-quinolone, termed *P**seudomonas* quinolone signal (PQS), binds to its cognate receptor PqsR regulating PQS biosynthesis and several virulence factors (6, 7). In this regulatory hierarchy, the levels of the PQS signal are controlled by both the Las and Rhl QS systems (8).

Within the host, several environmental cues feed into *the P. aeruginosa* QS networks, including host factors (9, 10) and essential nutrients, not least of which is iron (11). Iron acquisition is coordinated by a large number of genes activated upon iron starvation including the siderophores pyoverdine (PVD) and pyochelin (12), heme uptake through the heme assimilation system (*has*) and the *Pseudomonas* heme uptake (*phu*) system (13), and several extracytoplasmic σ factors that regulate many genes in response to iron (14). Iron starvation is known to induce the QS systems (15, 16), and conversely, several QS regulators including MvfR (PqsR) (17) and VqsR (18) have been shown to induce several iron-responsive genes.

The complex relationship between iron acquisition and QS-driven adaptation within the host is further complicated by the observation that there is an increased reliance on heme and ferrous iron at the expense of siderophore mechanisms in chronic infection. Previous gene expression and metabolism studies of *P. aeruginosa* longitudinal isolates from a CF lung demonstrated a significant reduction in siderophore production and enhanced utilization of heme (19). Furthermore, within-host genomic analysis of clinical *P. aeruginosa* strains revealed positive selection for mutations within the promoter region of the *phu* locus, resulting in increased expression of the PhuR receptor that coincides with a loss in PVD biosynthesis (20). Consistent with the Phu system being the major heme transport system in chronic infection, we have previously shown the Has and Phu systems serve non-redundant functions in heme sensing and uptake, respectively (21). However, extracellular heme internalized by either system is degraded by the iron-regulated heme oxygenase (HemO) releasing iron, carbon monoxide and the metabolites biliverdin IXβ (BVIXβ) and BVIXδ that are excreted to the extracellular environment (22). Utilizing the Δ*hemO* complemented strains Δ*hemO::hemO*α (hemOα modified to produce BVIXα) or Δ*hemO::hemO*in (catalytically inactive hemO), we determined the heme-dependent signaling cascade is post-transcriptionally regulated by BVIXβ and/or BVIXδ metabolites allowing for a rapid response to fluctuating extracellular heme levels (23). In keeping with their non-redundant function in heme uptake and sensing, the *has* genes were upregulated between 100- and 300-fold in an acute mouse lung infection model, whereas the Phu system was upregulated ~15- to 30-fold (24). Despite the fact the heme uptake systems may have distinct roles in acute and chronic infections, the regulatory mechanisms underlying how heme is integrated into the QS regulatory networks governing this transition is poorly understood.

QS is complex and responsive to numerous interconnected extracellular signals including cell density, secretion of small molecule effectors, as well as physical factors associated with surface attachment. Furthermore, two-component sensor systems allow *P. aeruginosa* to adapt and streamline their virulence factors in response to external stimuli such as QS via phosphorelay or c-di-GMP, a critical factor in coordinating extracellular and intracellular signal pathways (25). *P. aeruginosa* also encodes four distinct chemosensory systems that integrate extracellular signals from several chemoreceptors that respond to a variety of effectors such as oxygen and nitric oxide, amino acids, inorganic phosphate, polyamines, and organic acids (26). The coordination of these systems is critical to the transition from a planktonic to sessile lifestyle associated with *P. aeruginosa* biofilm formation and chronic infection. Herein, we constructed *hemO*α and *hemO*in allelic strains and show that the lack of endogenous BVIXβ and BVIXδ production in these strains results in dysregulation of QS, motility and biofilm formation, all strategies required for the transition from acute to chronic infection. Taken together, the data suggest that the physiological heme metabolites BVIXβ and/or BVIXδ function as signaling or effector molecules integrating the switch in iron acquisition systems with the central QS regulatory-driven adaptations that enhance fitness within the host.

## RESULTS

### HemO and its metabolites BVIXβ and BVIXδ are required for optimal growth of *P. aeruginosa*

**Fig 1:**
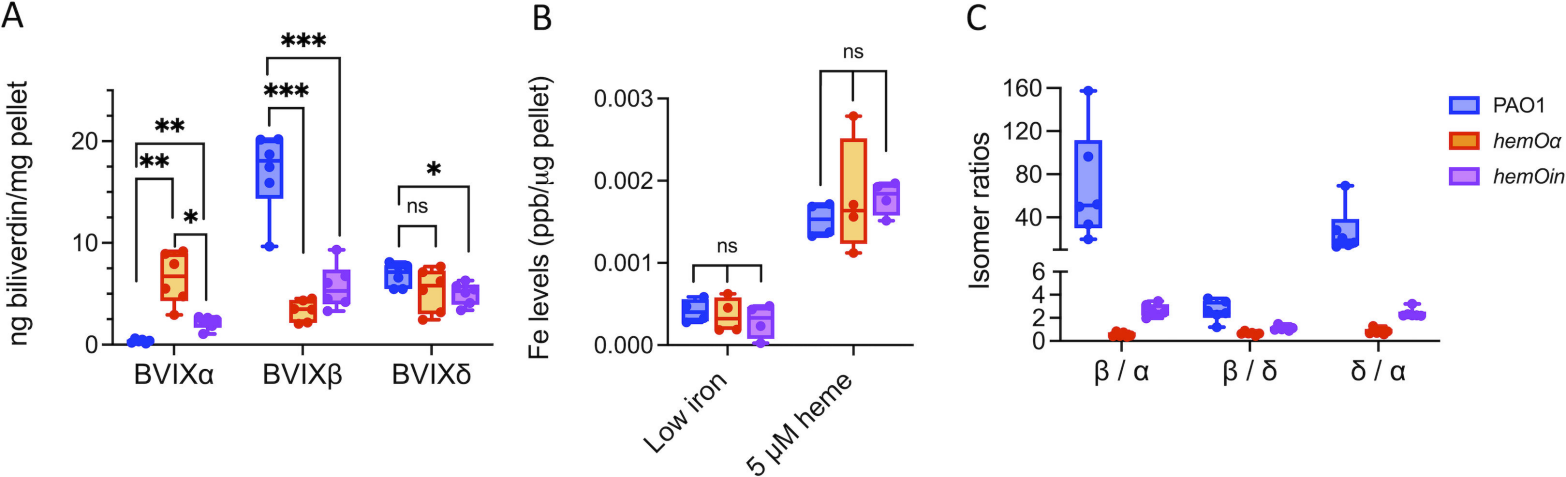
Biliverdin and iron levels in the *hemO* allelic strains compared to PAO1 in shaking conditions. (**A**) LC-MS/MS quantification of the BVIX isomers when supplemented with 5 µM heme. Cells were harvested at OD_600_ of 1 as described for the proteomics experiments. BVIX levels reported are those for the pellet and collected supernatant combined. BVIX values represent the standard deviation of *n* = 6. *P* values as determined by Student *t*-test comparing the BVIXβ and BVIXδ level isomers from the *hemO* allelic strains to PAO1 WT, or the BVIXα levels in the PAO1 WT and *hemO*in strains to *hemO*α, where *P < 0.05, **P < 0.005, and ***P < 0.001. (**B**) ICP-MS analysis of the intracellular iron levels in PAO1 WT and *hemO*α and *hemO*in allelic strains at 4 h. Values for each strain represent the average and standard deviation of *n* = 4. Strains and conditions as shown in the figure legend. (**C**) Ratio of BVIX isomers within the PAO1 and *hemO* allelic strains. ns, not significant; WT, wild type.

The allelic strains *hemOin* and *hemOα* were grown in minimal media supplemented with 5-µM heme as the sole iron source, growth conditions where we observed BVIX metabolites in the extracellular media (23). The *hemO* allelic strains showed a lag in growth compared to the parent PAO1 strain in the presence of heme (Fig. S1A). Although a lag in growth might have been expected for the *hemOin* strain lacking an active HemO to release iron, the HemOα enzyme has been shown to have similar activity to that of the wild-type (WT) enzyme (23). Moreover, the slow growth phenotype was observed in low iron conditions and was not rescued on supplementation with 5 µM FeCl_3_ (Fig. S1B). To confirm the slow growth was not due to an inability to efficiently utilize heme, we performed liquid chromatography-tandem mass spectrometry (LC-MS/MS) and inductively coupled plasma mass spectrometry (ICP-MS) to determine the levels of BVIX and iron, respectively. To accurately account for the total BVIX metabolite levels under the same conditions as the proteomics analysis, we collected cells at an OD_600_ of 1.0 (6 and 8 h to account for the difference in growth rate of the PAO1 WT and *hemO* allelic strains). BVIX levels were determined for the cell pellets and the collected supernatants following centrifugation to account for differences in excretion of the BVIX isomers to the media. As expected, in the PAO1 WT, the BVIXβ and BVIXδ isomers were predominant with only background levels of BVIXα (Fig. 1A). In contrast, the *hemO*α strain showed a statistically significant increase in BVIXα over the BVIXβ and BVIXδ isomers. For the *hemOin* strain, despite the HemOin being catalytically inactive, we detected all three isomers in similar amounts (Fig. 1A). Heme bound to heme proteins has been observed to undergo a slow, non-enzymatic degradation in the presence of a suitable reducing agent by a process known as coupled oxidation (27). Furthermore, the catalytic activity of HemO is required to drive extracellular heme uptake, and in its absence, heme in the medium is chemically degraded via secreted redox active molecules such as phenazines (PHZs), giving rise to all three isomers (23). Therefore, the non-enzymatic coupled oxidation of extracellular heme in cultures of the *hemO*in strain leads to non-discriminate production of all three BVIX isomers (Fig. 1A). This is evident when looking at the levels of the BVIX isomers in the pellet and supernatant, respectively (Fig. S2). For the PAO1 WT, the levels of BVIXβ isomer in the pellet are fivefold to sixfold greater than the supernatant, whereas the BVIXδ isomer is more evenly distributed, suggesting it is more readily excreted to the extracellular medium (Fig. S2A). Interestingly, for the *hemO*α strain, the BVIXα isomer is significantly higher in the supernatant than the pellet, suggesting it is more readily excreted to the media (Fig. S2B). In the *hemOin* strain, the higher levels of the physiological BVIXβ and BVIXδ isomers in the supernatant compared to the pellet are consistent with the extracellular degradation of heme (Fig. S2C). Furthermore, when plotting the ratio of the total BVIX isomers within each strain for the combined supernatant and pellet (Fig. 1C), a clear pattern emerges where the levels of the BVIXβ and BVIXδ isomers are significantly higher in the PAO1 WT strain compared to the *hemO* allelic strains. Interestingly, the iron levels are also similar in all three strains, from iron released enzymatically in the case of the PAO1 WT and *hemO*α strains, or active transport from the extracellular medium following coupled oxidation in the *hemO*in allelic strain (Fig. 1B). Taken together, the data suggest that the lag in growth is independent of iron and more likely a result of the lack of intracellular BVIXβ production in the *hemO*α and *hemOin* strains (Fig. S2).

### Comparative proteomics of the hemO allelic strains in shaking conditions reveals global changes in QS, virulence, motility, and biofilm formation

To determine the underlying factors behind the slow growth phenotype, we performed comparative proteomics analysis of the *hemO* allelic strains. The strains were grown in M9 media supplemented with 5 µM heme and grown to late exponential stage. Cells were harvested at an OD_600_ of 1.0 to compare cultures at similar growth stage and cell density (collected at 5 and 8 h to account for the lag in growth of the *hemO* allelic strains). Quantitative label-free proteomics was performed by nano ultraperformance liquid chromatography coupled to high-resolution tandem mass spectrometry as described in Materials and Methods. Protein levels that were significantly (*P* < 0.05, *n* = 5) changed at least twofold (equivalent to 1 log_2_ fold change) were considered further.

The *hemOα* allelic strain compared to PAO1 showed a significant increase in abundance of 194 proteins and a significant decrease in abundance of 96 proteins (Table S1). Similarly, for the *hemOin* strain, we observed a significant increase in abundance of 196 proteins and a significant decrease in abundance of 82 proteins (Table S1). We next performed network analyses using the Search Tool for the Retrieval of Interacting Genes/Proteins (STRING) database, which generates a map of biological connections between proteins that are upregulated or downregulated (28). The maps display the proteins as nodes connected by edges of known interactions based on experimental and predicted interactions. The networks created for the upregulated or downregulated proteins for the *hemOα* and *hemOin* strains following growth in the 5 µM heme showed significantly more interactions than a random collection of genes, indicating a significant biological relationship between the proteins in each data set (Table S2).

**Fig 2:**
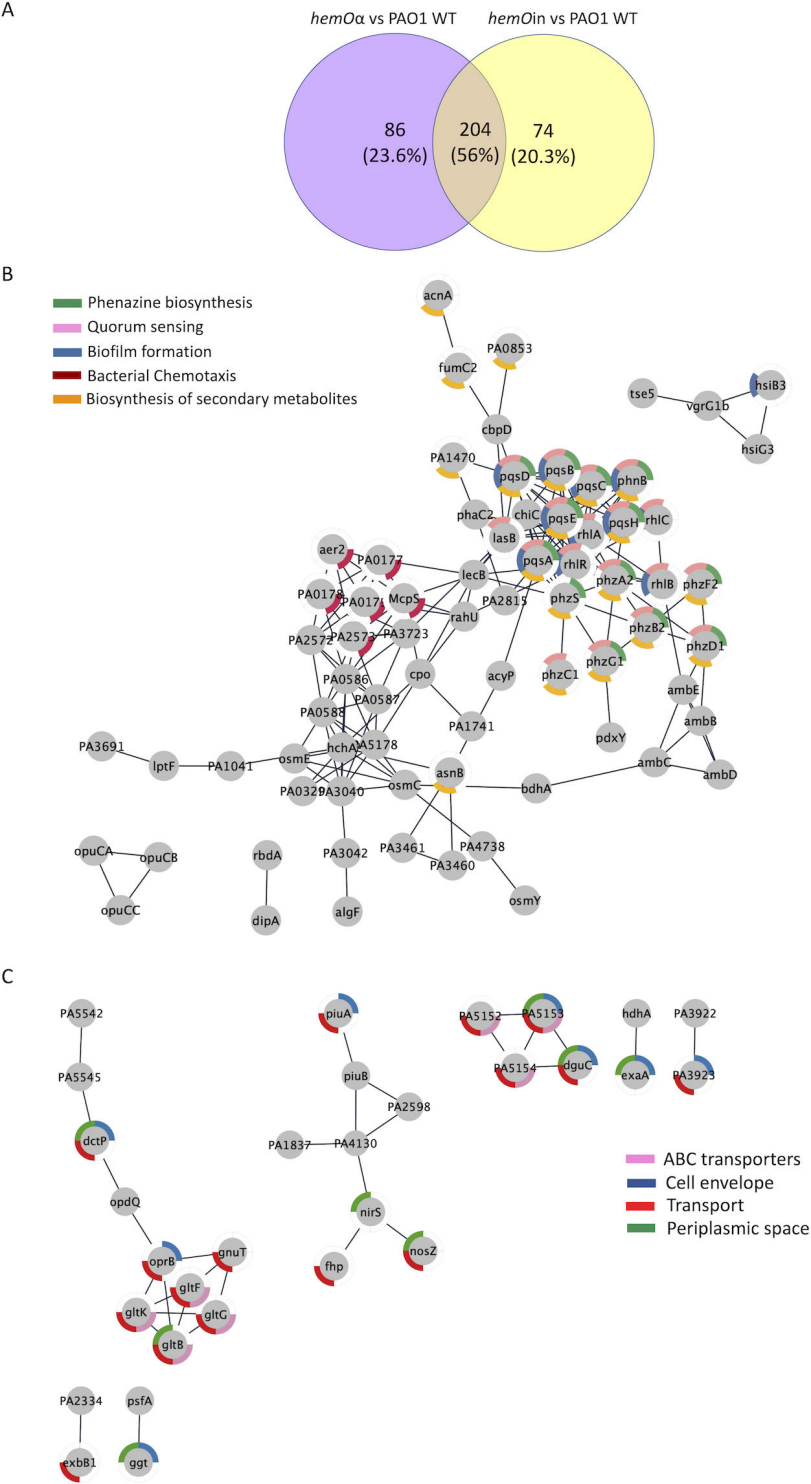
Comparison of the differentially regulated proteins common to the proteome of the *hemO*α and *hemO*in strains compared to PAO1 WT in shaking conditions. (**A**) Venn diagram of significantly (*P* < 0.05) downregulated and upregulated proteins common to the *hemO* allelic strains when supplemented with 5 µM heme. Proteomics was performed on an *n* of 3, and significance as determined by analysis of variance test corrected for multiple testing by applying a Benjamini-Hochberg procedure (34). Network analysis performed using the STRING database (28) on the proteins that were significantly upregulated (**B**) and downregulated (**C**) when supplemented with 5 µM heme. The network was transferred into Cytoscape (35), and the pathways were color coded as shown.

Interestingly, a comparison of the upregulated and downregulated proteins in the *hemOα* and *hemOin* strains revealed 56% were common to both with 146 upregulated and 54 downregulated proteins (Fig. 2A; Table S1). Network analyses of the upregulated and downregulated proteins common to both *hemO* allelic strains showed significantly more interactions than a random set of proteins (Table S3). A Kyoto Encyclopedia of Genes and Genomes pathway analysis of cellular activities and metabolic pathways significantly affected by the loss of endogenous BVIXβ and BVIXδ production showed an increase in proteins involved in phenazine production, QS, biofilm formation, and chemotaxis (Table 1). While the downregulated proteins were fewer, they were all involved in transport, particularly ATP-binding cassette transporters and proteins associated with the cell envelope and periplasmic space (Table 1). Moreover, network analysis of proteins common to both strains revealed a significant upregulation of proteins required for the synthesis of alkyl-4-quinolones (AQs) as well as several QS regulators and response proteins (Fig. 2B; Table S1). Specifically, a significant increase was observed for the 2-heptyl-3-hydroxy-4-quinolone (PQS) biosynthesis and signaling proteins (PqsA, PqsB, PqsD, PqsE, PqsH, and PqsL), as well as the transcriptional regulators LasR and RhlR (Fig. 2B; Table S1). We also observed an increase in several QS-regulated virulence factors including the pyocyanin (PYO) biosynthesis proteins (PhzA2, PhzB2, PhzC2, PhzC1, PhzD1, PhzG1, and PhzS), extracellular proteases (AprD and LasB), rhamnolipids (RhlA, RhlB, and RhlC), chitinase (ChiC), and lectins (LecB). Consistent with the upregulation of PQS biosynthesis, we observed that the global QS repressor QsrO was significantly downregulated (Table S1). Interestingly, most of the identified QS related proteins were those regulated by the PqsE-RhlR autoinducer system that is central to group behaviors such as biofilm formation (29). Moreover, we observed a significant upregulation in the phosphodiesterases (PDEs) DipA (30) and RbDA (31) associated with biofilm dispersal, as well as a number of methyl-accepting chemotaxis proteins involved in flagella-mediated motility, including the soluble PAS domain chemoreceptors PA1930 (McpS), PA2573, and PA0176 (Aer2) in the *hemO* allelic strains (Table S1). The Aer2/McpB (PA0176) chemoreceptor, along with its cognate sensor, transduction, and response regulator proteins (PA0177, PA0178, and PA0179), has previously been shown to be required for virulence (32). These chemoreceptors and biofilm dispersal proteins all belong to the PAS domain family of chemoreceptors and are most often associated with response to changes in oxygen, redox, or nutrients (33).

**TABLE 1:** Kyoto Encyclopedia of Genes and Genomes pathway analysis of differentially expressed proteins in the *hemO* allelic strains versus PAO1 in shaking conditions

Pathway/cellular activity	*P* value
Upregulated versus PAO1 WT	
Phenazine biosynthesis	1.38 × 10^−9^
Quorum sensing	1.08 × 10^−5^
Biofilm formation	3.3 × 10^−3^
Bacterial chemotaxis	4.46 × 10^−2^
Downregulated versus PAO1 WT	
Outer membrane-bounded periplasmic space	5.0 × 10^−4^
Cell envelope	5.0 × 10^−4^
Periplasmic space	2.7 × 10^−3^

The overall dysregulation in QS and motility is accompanied by the upregulation of several proteins associated with the response to osmolarity and oxidative stress (OsmC, OsmE, OsmY, Cpo, and lipotoxin F [LptF]) (36, 37) and the osmoprotective transporter proteins (OpuCA, OpuCB, and OpuCC) (Table S1) (38). Interestingly, LptF has been shown to be highly expressed in mucoid strains and protects against hypochlorite stress (37). In addition to LptF, chloroperoxidase Cpo and several other AlgU-regulated proteins required for cell envelope homeostasis were upregulated, including the operon encoding the chitin binding protein CbpD, oxidoreductase PA0853, and fumarate hydratase FumC2 (39). The core proteome common to both *hemOα* and *hemOin* strains is consistent with dysregulation of the PQS signaling pathway and several downstream target genes including those required for motility, biofilm formation, and maintenance.

A significant downregulation was observed for proteins associated with the cell envelope and periplasmic space (Table 1). These included energy-dependent pathways involved in glucose/gluconate (OprB, GltK, GltG, GltF, GltB, and GntP), C4-dicarboxlic acid (DctP), and arginine/ornithine uptake (PA5152–PA5154) (Fig. 2C; Table S1). Interestingly, we observed downregulation of denitrification proteins (NirS and NosZ) and the NO detoxifying dioxygenase (Fhp) (Fig. 2C; Table S1). The increased expression of proteins associated with cell envelope stress and QS is consistent with the slow growth phenotype and suggests that the lack of BVIXβ and BVIXδ causes a pleiotropic stress response in the *hemO* allelic strains.

### The *hemOα* and *hemOin* planktonic cultures have increased levels of AQs, homoserine lactone, and PHZs

**Fig 3:**
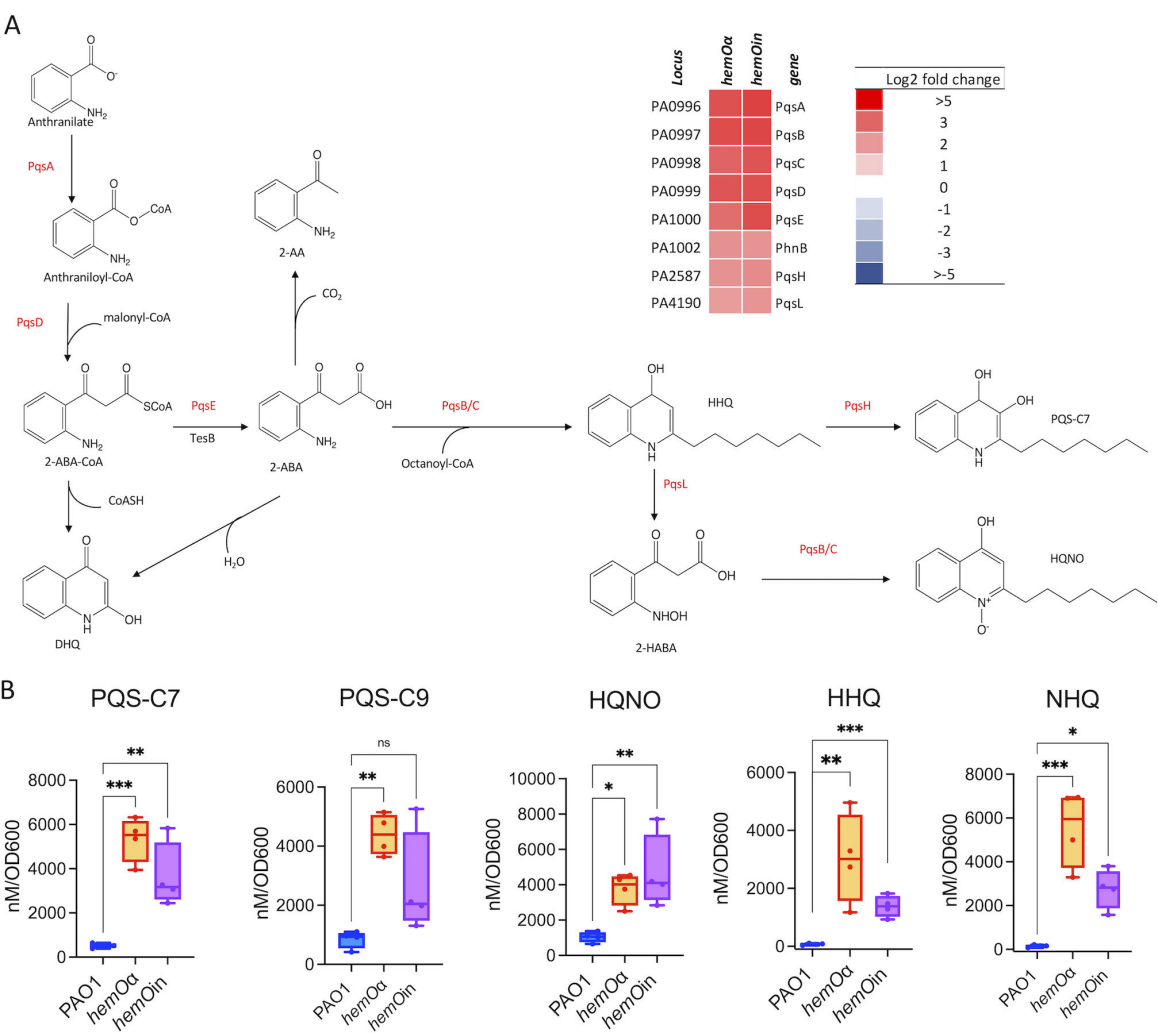
The quinolone-based quorum-sensing system PQS is increased in the hemO allelic strains compared to PAO1 WT in shaking conditions. (**A**) PQS biosynthesis pathway and heat map of the proteins differentially regulated in the *hemO* allelic strains compared to PAO1 WT. The log_2_ fold changes of significantly (*P* < 0.05) upregulated proteins in the PQS biosynthesis pathway are shown. (**B**) LC-MS quantification of NHQ, HHQ, HQNO, and PQS quorum-sensing molecules. Cells were harvested at an OD_600_ of 1 as described for the proteomics experiments. Extraction and analysis as described in Materials and Methods. Experiments show the standard deviation of *n* = 4. *P* values as determined by Student *t*-test comparing the *hemO* allelic strains to PAO1 WT, where *P < 0.05, **P < 0.005, and ***P < 0.001.

**Fig 4:**
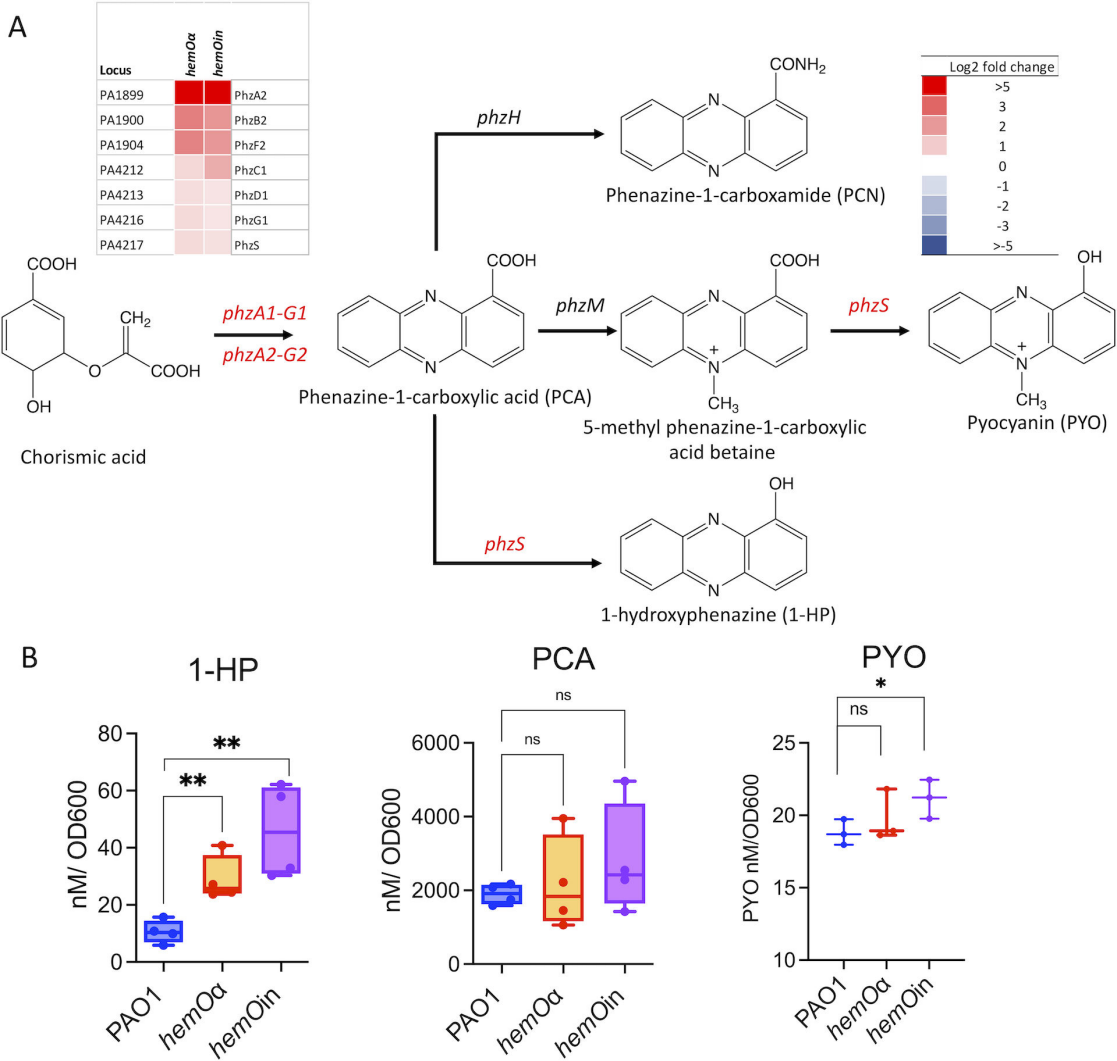
Phenazine biosynthesis is increased in the hemO allelic strains compared to PAO1 WT in shaking conditions. (**A**) Pyocyanin biosynthesis pathway and heat map of the proteins differentially regulated in the *hemO* allelic strains compared to PAO1 WT. The log_2_ fold change of significantly (*P* < 0.05) upregulated proteins in the PQS biosynthesis pathway are shown. (**B**) LC-MS and spectroscopic quantitation of 1-HP and PCA and PYO, respectively. Cells were harvested at an OD_600_ of 1 as described for the proteomics experiments. Extraction and analysis as described in Materials and Methods. Experiments show the standard deviation of *n* = 4. *P* values as determined by Student *t*-test comparing the *hemO* allelic strains to PAO1 WT, where *P < 0.05 and **P < 0.005.

Based on the proteomics analysis, we measured the levels of the secreted AQs for the *hemO* allelic strains compared to PAO1. The upregulation of proteins in the PQS biosynthesis pathway in both *hemO* allelic strains (Fig. 3A) led to increased levels of PQS-C7 and PQS-C9 as well as 4-hydroxy-2-heptylquinoline (HHQ) and 2-nonyl-4(1*H*)-quinolone (NHQ) (Fig. 3B). In addition, there were increased levels of the N-oxide derivative 4-hydroxy-2-heptylquinoline-*N*-oxide (HQNO) that has been shown to have autopoisoning activity toward the cytochrome bc1 complex by promoting autolysis or programmed cell death in concert with the generation of reactive oxygen species (40). Similarly, the increased expression of proteins involved in the QS-regulated phenazine biosynthesis pathway (Fig. 4A), particularly PhzS, was concomitant with increased production of 1-hydroxyphenazine (1-HP) in both *hemO* allelic strains (Fig. 4B). In contrast, the levels of phenazine-1-carboxylic acid (PCA) in both *hemO* allelic strains were similar to that of PAO1 WT (Fig. 4B). Although PYO was statistically significant in the *hemO*in strain compared to PAO1 WT, the physiological levels were very similar across all three strains (Fig. 4B). In both allelic strains, we also observed an increase in the LasR ligand 3-oxo-C12 homoserine lactone (HSL) consistent with the early onset of QS (Fig. S7A). As QS is central to group behaviors such as swarming and biofilm formation, we performed a series of motility and rhamnolipid analyses comparing the *hemO* allelic strains to the parent PAO1 strain.

### The *hemOα* and *hemOin* strains show altered motility and decreased biofilm formation

**Fig 5:**
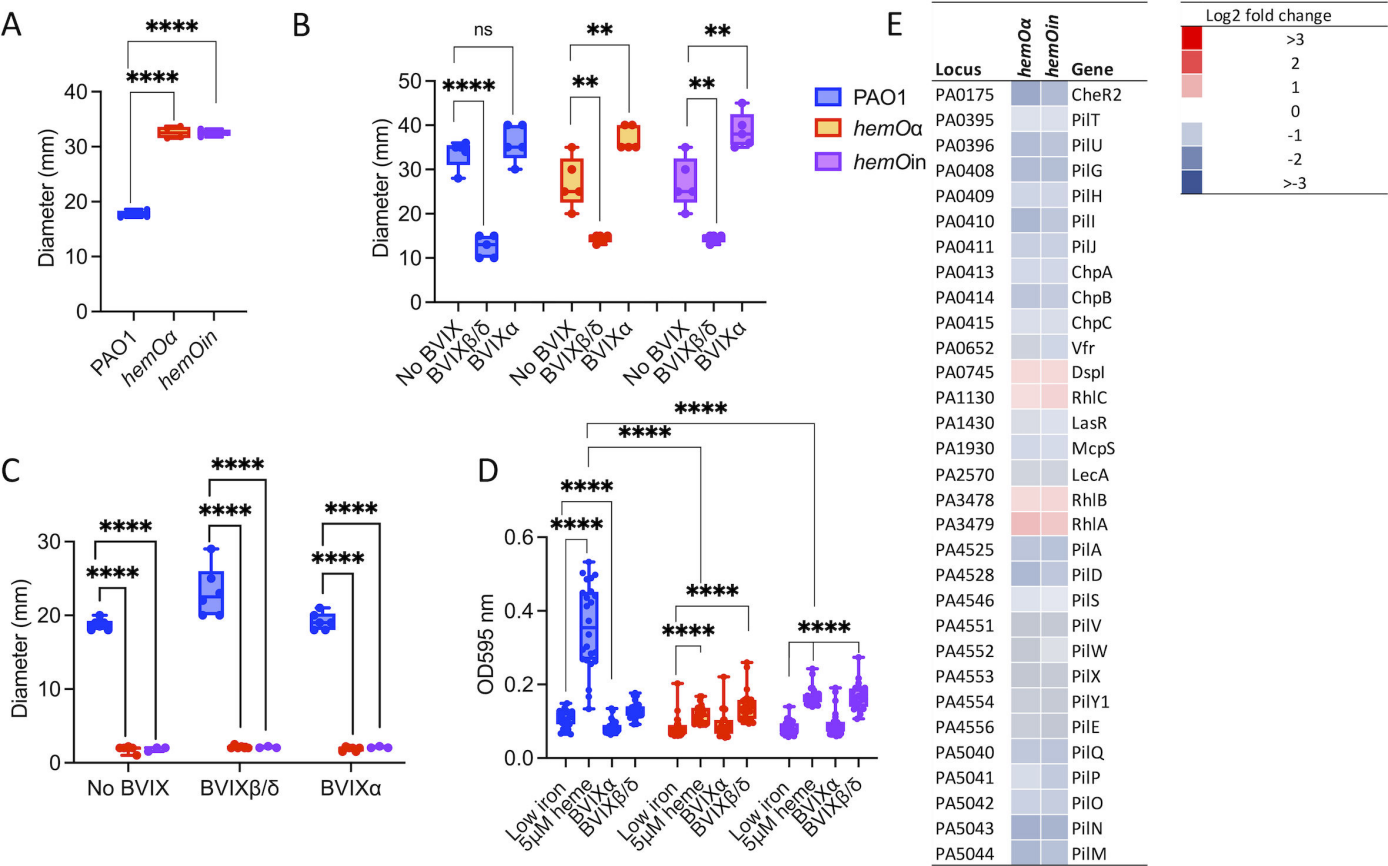
Motility and biofilm formation are disrupted in the hemO allelic strains compared to PAO1 WT. (**A**) Swimming motility assays. *P* values as determined Student *t*-test comparing the *hemO* allelic strains to PAO1 WT, where ****P < 0.0001. (**B**) Swarming motility assays in the presence and absence of a 5-µM mix of BVIXβ/δ or BVIXα. *P* values as determined by Student *t*-test comparing the BVIX-supplemented plates to the non-supplemented plates within the respective strains and between strains in the absence of BVIX, where **P < 0.005 and ****P < 0.0001. (**C**) Twitching motility assays in the presence and absence of a 5-µM mix of BVIXβ/δ or BVIXα. *P* values as determined by Student *t*-test comparing the BVIX-supplemented plates to the non-supplemented conditions within the respective strains and between strains in the absence of BVIX, where ****P < 0.0001. (**D**) Relative biofilm formation as determined by crystal violet assay for the strains supplemented with either 5-µM heme, BVIXβ/δ, or BVIXα. *P* values as determined by Student *t*-test comparing the BVIX-supplemented plates to the non-BVIX-supplemented conditions within the respective strains and between strains in 5-µM heme, where ****P < 0.0001. Motility and biofilm assays were performed as described in the Materials and Methods on a minimum of *n* = 6 for each strain. (**E**) Heat map of motility-related proteins differentially regulated in the *hemO* allelic strains compared to PAO1 WT. The log_2_ fold change of significantly (*P* < 0.05) upregulated or downregulated proteins are shown. ns, not significant.

Based on the differential expression of genes involved in QS, biofilm formation, and motility in shaking conditions, we further characterized the swimming, swarming, and twitching abilities of the *hemO* allelic strains, as well as their ability to form biofilms. The *hemOα* and *hemOin* strains demonstrated an increase in flagella-mediated swimming compared to PAO1 WT (Fig. 5A). In contrast, whereas there was no statistical difference in the diameter of swarming between the *hemO* allelic strains and PAO1, they displayed distinct phenotypes (Fig. 5B; Fig. S3). In PAO1 WT, we observed the characteristic dendritic phenotype associated with swarming, whereas for the *hemO* allelic strains, the surface spreading observed is more similar to sliding a phenotype typified by loss of flagella and/or T4P (Fig. S3) (41). Interestingly, addition of the BVIXβ and BVIXδ mix to the plates inhibited swarming in all three strains, whereas addition of BVIXα recovered swarming in the *hemO* allelic strains to that of PAO1 WT (Fig. 5B; Fig.S3).

We also observed an increase in rhamnolipid production in the *hemO* allelic strains (Fig. S4) which is consistent with the observed sliding phenotype (Fig. 5B; Fig. S2) and the upregulation of the biosynthesis proteins RhlA, RhlB, and RhlC in both shaking and static conditions (Tables S1 and S4). Rhamnolipids have been shown to be important effectors of biofilm structure where they have been proposed to play a role in microcolony formation, biofilm architecture, and dispersal (42, 43).

Perhaps most surprisingly, the *hemO* allelic strains were completely deficient in twitching when compared to PAO1 WT (Fig. 5C). On removing the agar, the *hemO* allelic strains were more easily detached from the plate than the PAO1 WT cells (Fig. S5). Addition of BVIXβ and BVIXδ to the agar increased adherence of both PAO1 WT and the *hemO* allelic strains to the plates (Fig. S5). In contrast, addition of BVIXα to the *hemO* allelic strains did not show a significant increase in either adherence or twitching radius compared to PAO1 WT (Fig. 5C; Fig. S5). As expected, given the motility and adherence defects, both *hemO* allelic strains when grown with 5 µM heme showed reduced biofilm formation compared to PAO1 WT (Fig. 5D). Addition of the physiological mix of BVIXβ and BVIXδ to the *hemO* allelic strains showed a slight but significant increase in biofilm mass, whereas addition of BVIXα to the strains did not show any physiologically relevant increase in mass (Fig. 5D). We attribute the increased biomass on addition of BVIXβ and BVIXδ to the observed increase in adherence (Fig. S5). In contrast to growth in 5 µM heme, supplementation with 5 µM FeCl_3_ gave less robust biofilm formation and no significant differences between PAO1 and the *hemO* allelic strains (Fig. S6), suggesting the BVIXβ and/or BVIXδ metabolites have a positive effect on biofilm formation independent of iron.

### Comparative proteomics of the hemO allelic strains in static conditions reveals significant changes in proteins involved in motility and biofilm formation

**Fig 6:**
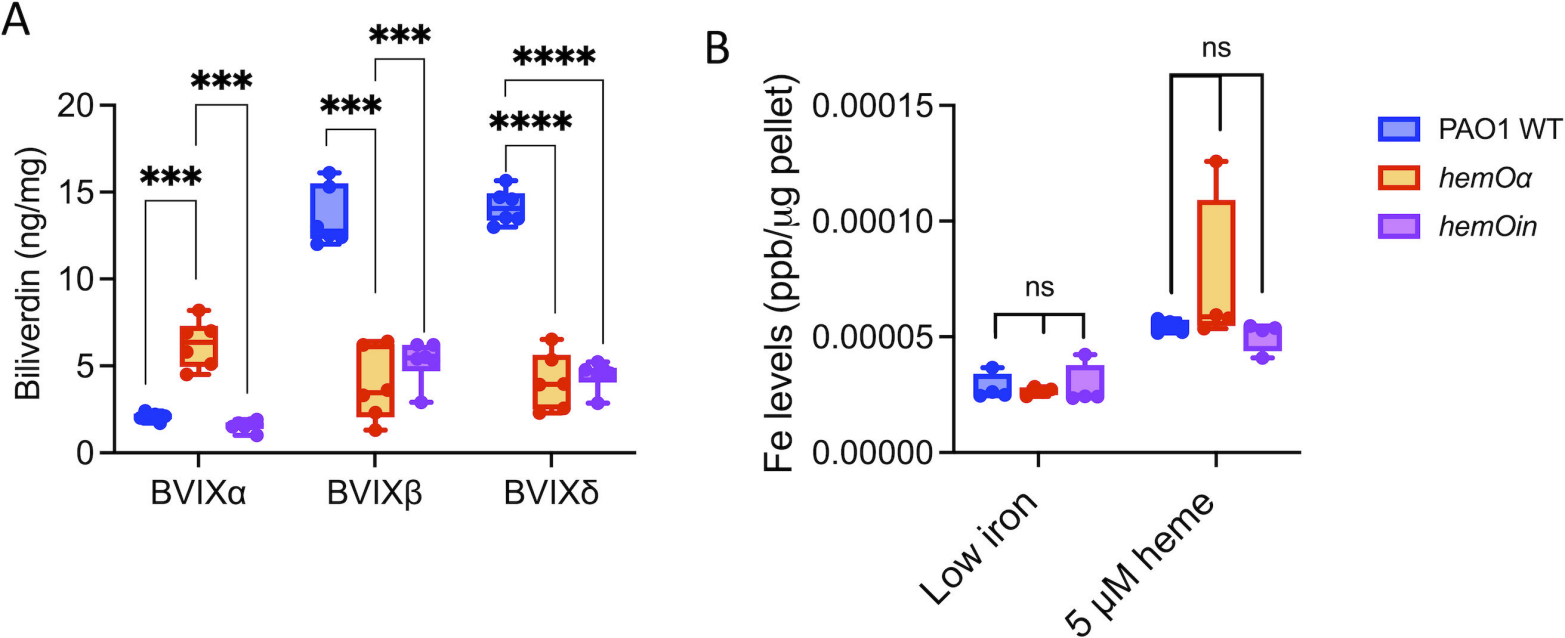
Biliverdin and iron levels in PAO1 WT and the hemO allelic strains in static conditions. (**A**) LC-MS/MS quantification of the biliverdin (BVIX) isomers when supplemented with 5 µM heme. BVIX values represent the standard deviation of six biological replicates. *P* values as determined by Student *t*-test comparing the BVIXβ- and BVIXδ-level isomers from the *hemO* allelic strains to PAO1 WT, or the BVIXα levels in the PAO1 WT and *hemO*in strains to *hemO*α, where ***P < 0.001 and ****P < 0.000.1. (**B**) ICP-MS analysis of the intracellular iron levels in PAO1 WT, *hemO*α, and *hemO*in allelic strains from cells following growth on plates for 16 h. Values for each strain and time point represent the average and standard deviation of *n* = 4.

As the *hemO* allelic strains manifest motility defects in swarming, twitching, and biofilm formation, we repeated the proteomic analysis of the *hemO* allelic strains in static conditions. As observed for shaking conditions, the *hemO* allelic strains when grown on M9 agar plates supplemented with 5 µM heme showed a significant decrease in the physiological BVIXβ and BVIXδ isomers (Fig. 6A). Furthermore, the *hemO*α strain showed a statistical increase in BVIXα over both PAO1 WT and the *hemO*in strains. Similar to growth in shaking conditions, we did not observe a significant difference in iron levels between the *hemO* allelic strains and PAO1 wild type, suggesting that, even in static conditions, the *hemO*in strain can access iron via external heme degradation (Fig. 6B).

Comparative proteomics of the *hemOα* strain under static conditions revealed 521 differentially regulated proteins with 313 downregulated and 208 upregulated proteins (Table S4). Similarly, the *hemOin* strain showed differential regulation of 615 proteins with 374 downregulated and 241 upregulated proteins (Table S4). STRING analysis again showed significantly more interactions than a random collection of genes, indicating a significant biological relationship between the proteins in each data set (Table S5).

**Fig 7:**
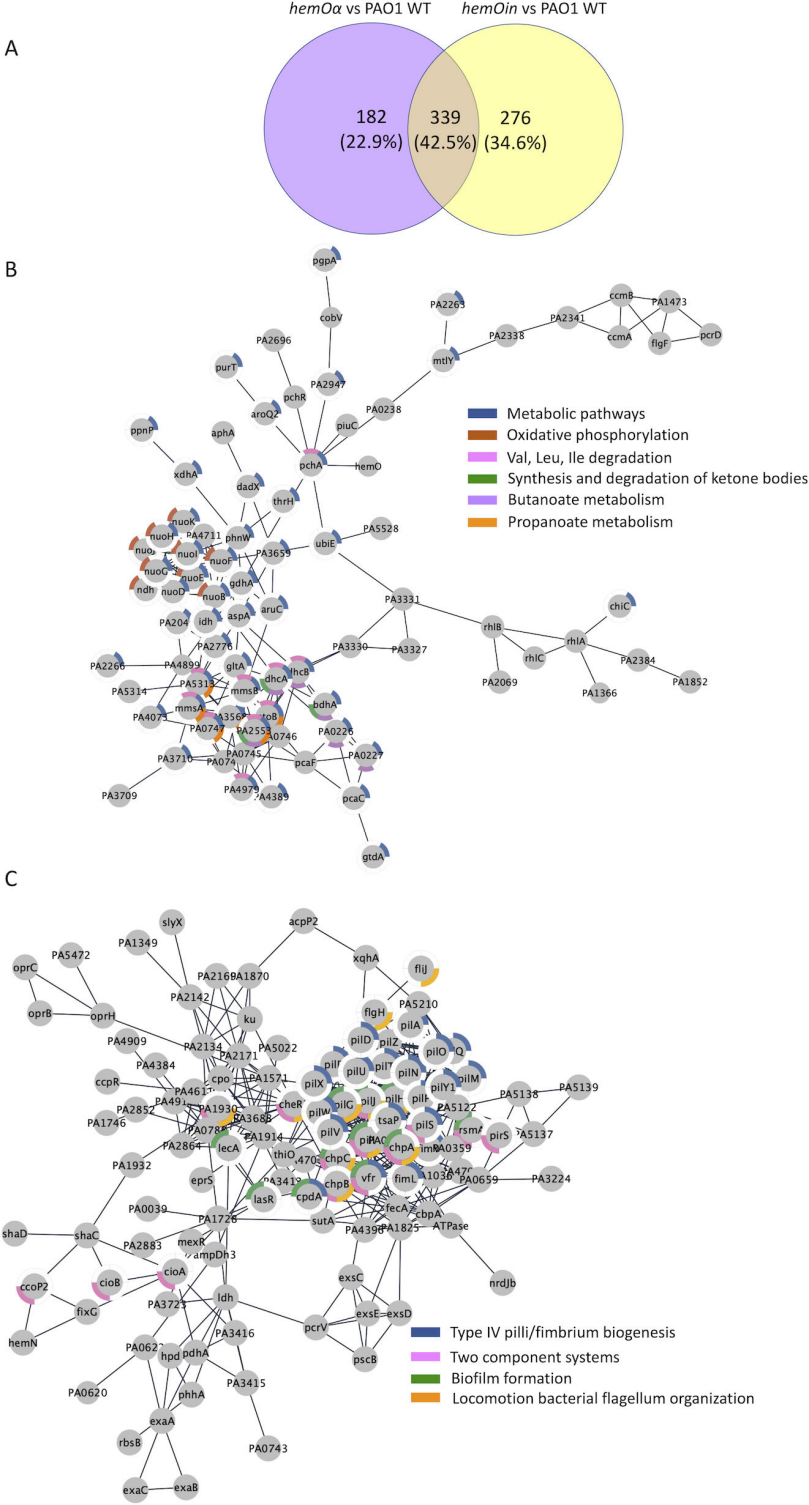
Comparison of the differentially regulated proteins common to the proteome of the hemOα and hemOin strains compared to PAO1 WT in static conditions. (**A**) Venn diagram of significantly (*P* < 0.05) downregulated and upregulated proteins common to the *hemO* allelic strains when supplemented with 5-µM heme. Proteomics was performed on an *n* of 3 and significance determined by analysis of variance test corrected for multiple testing by applying a Benjamini-Hochberg procedure (34). Network analysis performed using the STRING database (28) on the proteins that were significantly upregulated (**B**) and downregulated (**C**) when supplemented with 5-µM heme. The network was transferred into Cytoscape (35), and the pathways were color coded as shown.

Similar to growth in planktonic conditions, the number of differentially regulated proteins common to both *hemOα* and *hemOin* was 43% with 128 upregulated and 203 downregulated proteins (Fig. 7A; Table S4). Additionally, there are seven proteins (PA821, PA975, PA1054, PA2510, PA3285, PA3550, and PA3845) common to both strains that are differentially regulated (Table S4). Network analyses of the upregulated and downregulated proteins common to both strains again showed significantly more interactions than a random set of proteins, indicating a significant biological relationship between proteins in the data set (Table S3). Upregulated proteins affected by the loss of BVIXβ and IXδ as metabolites included an increase in proteins involved in metabolism, most notably, branched-chain amino acid degradation, oxidative phosphorylation, and the synthesis and degradation of ketone bodies (Table 2). These included proteins involved in branched-chain amino acids (Val, Ile, and Leu), propanoate and butanoate metabolism (MmsA, MmsB, Ldh, AtoB, DchA, DchB, GabT2, and LiuB), pathways that all feed into the tricarboxylic acid cycle at the level of acetyl-CoA or succinyl-CoA, respectively (Fig. 7B; Table S4). In addition, we observed changes in oxidative phosphorylation with an increase in proteins of the NADH dehydrogenase complex I (NuoBDEFGHIJK and Ndh) and a downregulation of proteins in the cytochrome c oxidase complex (CcoP2 and CcpR) and cyanide-insensitive terminal oxidases (CioA and CioB). The NADH dehydrogenase I complex ability to recycle NAD+ to NADH while generating a proton motive force for ATP production is consistent with the increased reliance on amino acid and fatty acid catabolism. The data suggest the *hemO* allelic strains adapt their energy metabolism in response to decreased endogenous BVIXβ/δ metabolite production, further suggesting these molecules may play a role as secondary metabolites in the growth and fitness of *P. aeruginosa*. In contrast to shaking conditions, we did not observe a significant increase in the PQS and PHZ biosynthesis proteins (Table S4). Furthermore, metabolite analysis revealed that the PQS metabolites were similar across all strains, except for HQNO, where the profile was reversed from shaking conditions with levels being significantly lower in the *hemO* allelic strains compared to PAO1 WT (Fig. S7C). The PHZ metabolites PCA and 1-HP also showed a profile similar to that observed in shaking conditions with 1-HP being significantly higher in the *hemO* allelic strains when compared to PAO1 (Fig. S7B).

**TABLE 2:** Kyoto Encyclopedia of Genes and Genomes pathway analysis of differentially expressed proteins in the *hemO* allelic strains versus PAO1 in static conditions

Pathway/cellular activity	*P* value
Upregulated versus PAO1 WT	
Metabolic pathways	1.51 × 10^−16^
Valine, leucine, and isoleucine degradation	5.16 × 10^−7^
Oxidative phosphorylation	1.32 × 10^−6^
Synthesis and degradation of ketone bodies	2.70 × 10^−5^
Butanoate metabolism	9.35 × 10^−5^
Propanoate metabolism	2.22 × 10^−3^
Downregulated versus PAO1 WT	
Pilus/fimbria biogenesis	3.86 × 10^−16^
Biofilm formation	6.20 × 10^−4^
Two-component systems	2.40 × 10^−3^
Taxis/locomotion/flagellum organization	4.80 × 10^−3^

Perhaps not surprisingly, given the defects in motility, the downregulated proteins were largely associated with biofilm formation, chemotaxis, and two-component systems (Table 2). Consistent with the downregulation in two-component systems and biofilm formation, STRING analysis identified several overlapping biological processes and pathways including those involved in chemotaxis and motility (Fig. 7C; Table S4). Consistent with the defect in twitching (Fig. 5C), the *hemO* allelic strains showed a significant downregulation in the TFP assembly (PilZ, PilA, PilD, PilV, PilW, PilX, PilY1, PilE, PilP, PilQ, PilO, PilN, PilM, PilT, PilU, and FimV) and the chemosensory pilus (Chp) (ChpA, ChpB, and ChpC) and associated proteins (PilG, PilH, PilI, and PilJ) critical for adherence, motility, and pathogenesis (Fig. 5E) (44, 45).

Interestingly, in contrast to shaking conditions, we see a downregulation of chemoreceptors associated with modulation of the Che chemosensory pathway including the cytosolic receptor McpS (PA1930) that negatively effects chemotaxis and clustering of chemoreceptors (46), PctC that responds to histidine, proline, and non-amino acid ligands such as γ-aminobutyrate (47), as well as an upregulation in the biofilm dispersion locus A BdlA soluble chemoreceptor that positively regulates biofilm dispersal through modulation of c-di-GMP levels and dispersion inducer DspI required for the biosynthesis of the diffusible signaling molecule cis-2-decenoic acid (48, 49). A downregulation of the biofilm initiation response sensor BfiS combined with the increase in proteins associated with dispersal is consistent with the less robust biofilm formation in the *hemO* allelic strains (Fig. 5D). We also observed a decrease in the CheR_2_ methyltransferase that specifically recognizes the Aer2/McpB soluble chemoreceptor of the Che2 chemosensory pathway (32). The Che and Che2 pathways form distinct arrays and respond to separate chemoreceptors and signals essential for not just chemotaxis but also virulence (32). Interestingly, the difference in regulation of the chemosensory pathways in shaking versus static reflects both the change in environmental signals such as aeration and surface tension and the timescale of bacterial growth. However, the significant effect on chemotaxis pathways involved in cooperative behaviors, adaptation, and virulence in both shaking and static conditions suggests that the utilization of heme as an iron source is integrated into the adaptation of *P. aeruginosa* from a planktonic to sessile lifestyle.

### Electron microscopy and Western blot of the hemO allelic strains show a decrease in surface piliation compared to PAO1

**Fig 8:**
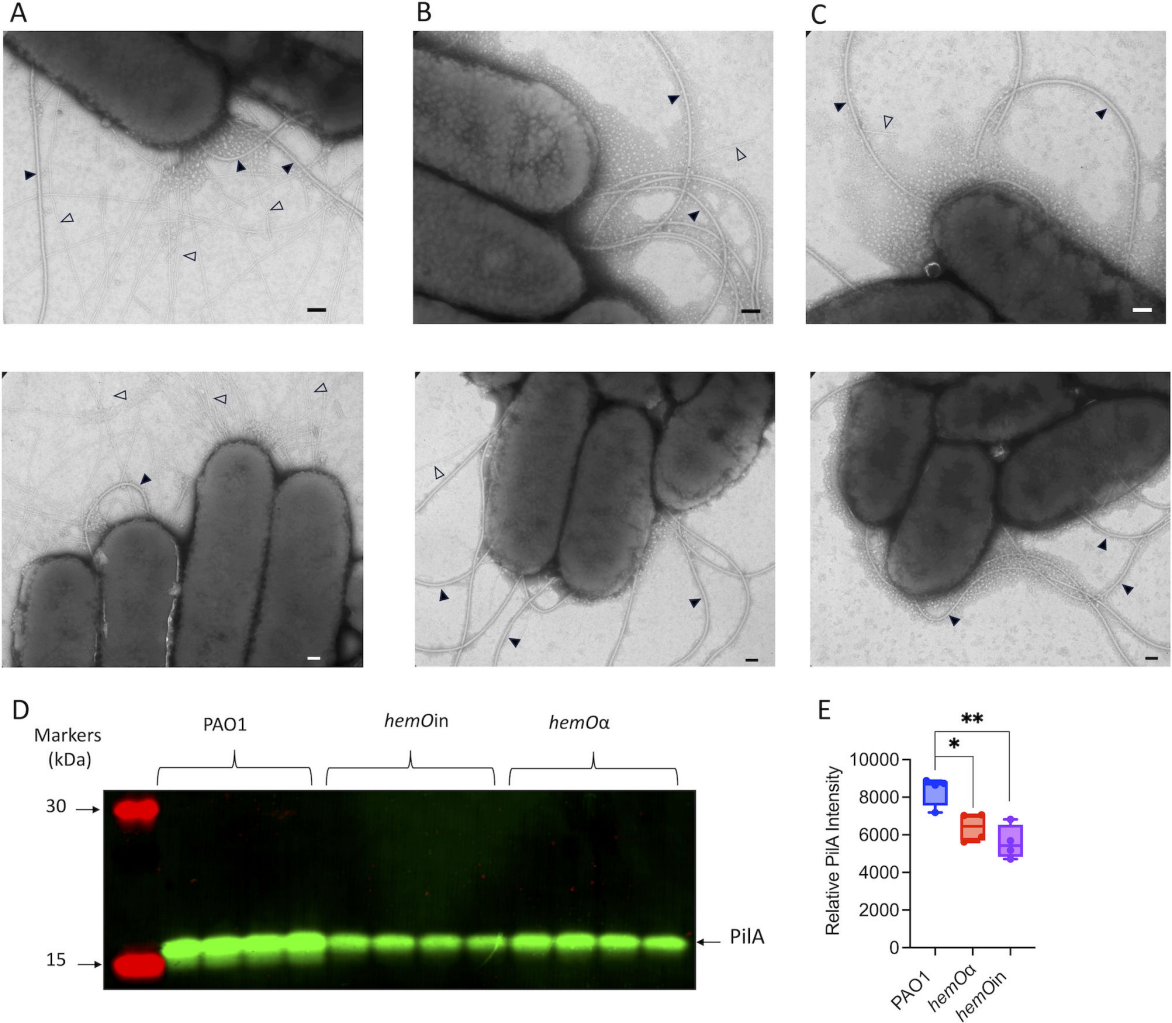
TEM and Western blot images of PAO1 WT and hemO allelic mutants. Electron micrographs of negatively stained PAO1 WT (**A**), *hemO*α (**B**), and *hemO*in (**C**) taken with an FEI tecnai T12 transmission electron microscope at 80 KV with an AMT bottom-mount camera. Upper and lower panels ×15,000 and ×11,000 magnification, respectively. Filled arrows highlight flagella and open arrows highlight pili. Bars = 100 nm. (**D**) Representative Western blot of PilA for the PAO1 WT and *hemO* allelic strains (left panel). (**E**) Relative intensity of PilA normalized to total protein. PilA relative intensity represents the average of *n* = 4. *P* values as determined by Student *t*-test comparing relative PilA intensity of the *hemO* allelic strains compared to PAO1 WT, where *P < 0.05 and **P < 0.005.

Given the twitching deficient phenotype and decrease in both the major (PilA) and minor (PilV, PilW, PilX, and PilE) pilins required for twitching, we analyzed the surface piliation by transmission electron microscopy (TEM) (50). The *hemO* allelic strains compared to the PAO1 WT strain showed a significant decrease in the total number of pili (Fig. 8A through C). Furthermore, the few pili that could be seen in the allelic strains were significantly shorter (~500 nm) than those in the PAO1 WT strain (~1 to 2 µm). Western blot analysis of PilA as a measure of surface piliation clearly showed a decrease in the *hemO* allelic strains compared to PAO1 WT (Fig. 8D and E).

## DISCUSSION

The adaptation of *P. aeruginosa* from a motile to sessile lifestyle characteristic of chronic infection requires the coordinated regulation of virulence factors by the QS network of diffusible signaling molecules regulating several virulence factors (2, 3). A host of environmental cues feed into *the P. aeruginosa* QS networks including host factors (9) and essential nutrients including iron (11, 17). The specific response to the limiting extracellular iron availability associated with infection is in part governed through the action of several extra cytoplasmic function sigma factors that sense the presence of the exogenous ferric-siderophore PVD or heme through their outer membrane receptors activating the cell surface signaling system and subsequent transcriptional upregulation of responsive genes, most often the outer membrane receptors for active uptake of the iron complex (14, 51). *P. aeruginosa* adapts to utilize specific iron sources, depending on the type of infection (acute versus chronic), and in host evolution leads to a switch to heme and ferrous iron utilization at the expense of the siderophore PVD (19, 20). The regulatory mechanisms by which this switch from ferric siderophore to heme utilization is mediated are not well understood but coincide with the switch from a planktonic to sessile lifestyle associated with biofilm formation and chronic infection. The current study suggests that independent of iron, the HemO specific heme metabolites BVIXβ and BVIXδ are critical secondary metabolites integrated into the regulatory networks controlling cooperative behaviors associated with chronic infection.

There are well-documented examples of the role of BVIXα as a signaling molecule in plants, algae, cyanobacteria and other bacterial species where it functions directly as a chromophore or precursor for the more reduced phycocyanobilin that can activate signaling mechanisms such as phosphorelay or second messenger signaling (52–54). Moreover, *P. aeruginosa* encodes a bacteriophytochrome, *bphP*, immediately downstream of a gene encoding a BVIXα-dependent heme oxygenase, BphO (55). Several lines of study have shown the *bphOP* operon is expressed during the stationary phase in a QS-dependent manner (56, 57). A recent study has further proposed AlgB is the response regulator of the BphP kinase integrating sensory responses to light with those of QS (58). Interestingly, isotopic ^13^C-heme labeling studies from our laboratory have shown that extracellular heme acquired by *P. aeruginosa* is exclusively shunted through the iron-regulated HemO by the cytoplasmic heme-binding protein, PhuS (59). Therefore, the differential expression and metabolic profiles of BphO and HemO, together with the alternate regioselectivities of the enzymes, support a distinct but separate role for the BVIX isomers in cell signaling. Furthermore, previous characterization of the *hemO*α and *hemOin* allelic strains, both of which lack the ability to endogenously produce BVIXβ and BVIXδ, allowed us to establish their role as regulatory molecules in the post-transcriptional upregulation of the extracellular hemophore, HasAp (23).

A predominant theme in the current study is that in both planktonic and static growth conditions, we observed in the *P. aeruginosa hemO* allelic strains a differential regulation of pathways involved in cooperative behaviors associated with the switch from a motile to sessile lifestyle. In shaking conditions, the slow growth phenotype coupled with the increased production of PQS might indicate the *hemO* allelic strains are sensing iron starvation, given the integration of HHQ and PQS cell-cell signaling with iron homeostasis (17), as well the ability of secreted PQS to trap iron (60). However, the *hemO* allelic strains are not iron starved as the intracellular iron levels are similar in all strains. Furthermore, the proteomics analysis does not indicate any increase in proteins associated with iron or heme acquisition as would be the case if the cells were sensing iron starvation (Tables S1 through S4). Increased levels of PQS have been shown to play a role in cooperative behavior to external stressors having both deleterious autolytic effects in programmed cell death as well as positive effects in transitioning surviving cells to a less active metabolic state (61). This pro- and antioxidant effect of PQS segregates the population, allowing for the survival of the more stress-tolerant cohort and shaping the overall bacterial population. The physiological effects of increased PQS are consistent with the slower growth phenotype observed for the *hemO* allelic strains (Fig. S1). Interestingly, the increase in PqsE-RhlR-dependent virulence factors including phenazines, secreted proteases, and rhamnolipids, as well as proteins required for oxidative and osmotic stress protection, further suggests the *hemO* allelic strains or a subpopulation is less able to adapt to external conditions. Furthermore, the upregulation of the chemosensory receptors associated with motility and virulence, and the observed increase in swimming motility suggests the cells are in a “hyper-motile” state. Moreover, we also observed upregulation of proteins involved in biofilm dispersal that have been shown to manifest distinct protein expression profiles from both planktonic and biofilm populations (62, 63). Collectively, the data suggest the *hemO* allelic strains are manifesting dysregulation in several pathways related to adaptation from a motile to sessile lifestyle.

In static conditions, the significant decrease in swarming and TFP-dependent twitching by the *hemO* allelic strains (Figure 7) is consistent with the downregulation of the TFP pili biogenesis and associated Chp chemosensory proteins (Figure 5). Specifically, we observed a downregulation of the outer membrane secreting PilQ, the pilus fiber PilA, and minor pilins PilVWX, as well as the PilG, PilJ, and ChpA proteins known to be required for increased piliation and chemotaxis (64–66). TEM confirmed the significant decrease in the number of pili in the *hemO* allelic strains compared to PAO1 (Fig. 8). Furthermore, the upregulation of rhamnolipid biosynthesis (Fig. S4) combined with the decrease in surface piliation (Fig. 8) most likely accounts for the distinct differences in the swarming profiles of the *hemO* allelic strains which are more typical of sliding rather than swarming motility (Fig. S3).

The downregulation of the TFP signaling machinery, together with the decrease in several chemoreceptors, is consistent with the dysregulation in motilities. TFP-mediated surface exploration by *P. aeruginosa* initiates a signaling cascade through the Chp chemosensory system leading to changes in cAMP and c-di-GMP levels required for adherence and early biofilm formation (44). Specifically, surface sensing by TFP leads to a PilJ mechanosensory-dependent increase in cAMP levels required for TFP biogenesis (67). This stimulation in cAMP and TFP production upregulates PilY1 an adhesin that impacts the surface-associated production of c-di-GMP as well as virulence (67). Hence, the downregulation of the TFP machinery, along with the upregulation of several PDEs associated with lowering c-di-GMP and inducing biofilm dispersal, would attenuate the defect in biofilm formation observed in the *hemO* allelic strains.

Furthermore, it is interesting to speculate, given the effect of exogenous BVIX isomers on adherence and biofilm formation, that one or both isomers may be ligands for a known or uncharacterized chemoreceptor. Interestingly, we have recently identified a BVIXβ-dependent helix-turn-helix transcriptional regulator we have termed biliverdin-dependent regulator specific for BVIXβ (BdrB) by thermal proteome profiling (C. Ladipo et al., unpublished data). Initial studies have shown the Δ*bdrB* strain has a similar twitching-deficient phenotype to the *hemO* allelic strains suggesting a direct regulatory link between heme metabolism and TFP-mediated motilities. Transcriptional regulation of chemoreceptor pathways is extremely complex, given the strict requirement to regulate a complex array of external signals that allow the bacteria to efficiently coordinate their response to optimize resources. Several transcriptional regulators, two-component systems, as well as several sigma factors function to activate or repress chemoreceptors and their chemosensory pathways. How the BVIX isomers might function in this complex chemosensory network of chemical stimuli is not known but is the subject of future studies.

In summary, we provide the first evidence that the unique *P. aeruginosa* HemO metabolites (BVIXβ and BVIXδ) act as signaling molecules integrating the utilization of extracellular heme as an iron source into the regulatory and chemosensory systems associated with adaptation from a motile to sessile lifestyle. The integration of the products of heme metabolism into the transcriptional and post-transcriptional regulation of cooperative behaviors associated with chronic infection provides a mechanism by which the cell can distinguish and adapt specifically within the host as a function of iron source. We propose to perform a transcriptomic and proteomic profile of the *hemO* allelic strains in biofilms as a first step toward understanding the global effects of heme metabolism on the switch from a planktonic to sessile lifestyle. Our current findings, taken together with previous studies showing in-host evolution to utilize heme as an iron source of *P. aeruginosa* chronic infection, highlight heme utilization as a viable therapeutic target for the treatment of such chronic infections.

## MATERIALS AND METHODS

### Bacterial strains

All bacterial strains, plasmids, and oligonucleotides used in this study are listed in Tables S6 and S7. *Escherichia coli* strains were routinely grown in Luria-Bertani (LB) broth (American Bioanalytical) or on LB agar. *P. aeruginosa* strains were freshly streaked and maintained on *Pseudomonas* isolation agar (PIA) (BD Biosciences). All strains were stored at −80°C in LB broth with 20% glycerol. For all proteomics, gene expression, and metabolism studies, a single *P. aeruginosa* WT or variant colony was picked and grown overnight in 10 mL of LB broth at 37°C. Cells were harvested by centrifugation and washed in 10 mL of M9 minimal media (Teknova). Following centrifugation, pelleted cells were resuspended in 10 mL of M9 minimal media and were used to inoculate 30 mL of fresh M9 media to a starting OD_600_ of 0.05. The cultures were grown at 37°C shaking for 3 h prior to induce iron limitation, after which either 5 µM heme (Frontiers Scientific) or FeCl_3_ was added. Shaking cultures were grown and samples were taken at various time points as specified for the proteomics, gene expression, and metabolite experiments (see below). For static cultures, LB overnight cultures were diluted 1 in 1,000 in M9 media, and 25 µL of the diluted culture was plated onto M9 agar supplemented with 5 µM heme and incubated overnight at 37°C. Heme solutions were prepared as previously described (23).

### BVIX isomer standards

Purified BVIXα was obtained from Frontier Scientific. The BVIXβ and BVIXδ isomers were produced by overexpression of HemO in a reengineered *E. coli* Nissle T7 strain as previously described (68). Briefly, *E. coli* Nissle T7 was transformed with the pET*hemO* by electroporation. Following growth on LB agar plates containing 100-µg/mL ampicillin (Amp) single colony was picked and grown overnight at 37°C in 10 mL LB-Amp and used the following day to inoculate 4 × 1 L LB-Amp flask to a final OD_600_ of 0.05. Protein production was induced at an OD_600_ of 0.5 on the addition of isopropyl β-D-1-thiogalactopyranoside to a final concentration of 1 mM grown for a further 2 h after which heme was added to a final concentration of 10 µM. Cultures were grown at 25°C for a further 12 h after which the cells were harvested, and the supernatant and pellets were stored at −80°C until further use. Extraction of the BVIX isomers from the supernatant was performed as previously described (68). BVIX isomers from the pellets were extracted as described for LC-MS/MS analysis (see below) and subjected to an extra purification step over a C18 Sep-Pak column (35 cc, Waters) as previously described (68). The BVIX isomers were dried down and stored under nitrogen in dark vials at −80°C.

### Construction of allelic mutant strains PAO1 *hemOα* and *hemOin*

*Pseudomonas* mutant strains *hemOα* and *hemOin* were obtained by allelic exchange as previously described (69) using the parental strain PAO1 Δ*hemO* (23). Briefly, a 0.7-kb fragment corresponding to *hemO* upstream sequence was PCR amplified from PAO1 chromosomal with primer pair *BamH*I-*hemO*-A/*EcoR*I-P*hemO*-R (Table S6). Following *BamH*I-*EcoR*I digestion, the amplified fragment was inserted into the previously described mini-CTX1-*hemO*α and mini-CTX1-*hemOin* plasmids (23). Similarly, the 0.7-kb *hemO* downstream region was PCR-amplified with primers *BseR*I-3′*hemO* and *Hind*III-3′*hemO*-R, digested, and cloned into *BseR*I/*Hind*III sites of the new mini-CTX1-5*′hemO*α and mini-CTX1-5*′hemOin* constructs to give the resulting mini-CTX1-5*′hemO*α3′ and mini-CTX1-5*′hemO*in3′. Finally, the fragment, including each *hemO* mutant allele plus their flanking regions, was obtained following *BamH*I-*Hind*III digestion and subcloned into the counter-selective suicide plasmid pEX18Tc (69). Final constructs pEX18Tc-5*′hemO*α3′ and pEX18Tc-5*′hemO*in3′ were confirmed by DNA sequencing (Eurofins Genomics LLC) and transferred from *E. coli* S17-1-λpir into *P. aeruginosa* Δ*hemO* by conjugation. A double event of homologous recombination followed by selection on PIA plates containing 5% sucrose resulted in the chromosomal integration of the *hemO*α or *hemO*in allele into the parental Δ*hemO* strain. To verify the correct allelic exchange event, the *hemO* gene was PCR-amplified with primers HemO-F/HemO-R and confirmed by sequencing.

### Swimming, swarming, and twitching motility assays

Swimming motility assays were performed as previously described with slight modification (70). Briefly, plates were prepared in M8 based media supplemented with 0.5% Casamino aAcids, 0.2% glucose, 1 mM MgSO_4_ to a final concentration of 0.3% agar. Approximately 25 mL of agar medium (0.5% agar) was poured into each plate and allowed to solidify at room temperature for 3–4 h before inoculation. A sterile P200 pipette tip was dipped into a 100-µL aliquot of the respective overnight bacterial LB cultures and used to stab the inoculate into the middle of the agar plate. The plates were incubated upright at 37°C for 24–48 h. Motility was assessed and quantified by measuring the diameter from the point of inoculation to the furthest point on the plate.

Swarming motility assays were adapted as previously described with some modifications (71). Briefly, the swarming plates were prepared as described above with M8 media containing 24 mM KH_2_PO_4_, 48 mM Na_2_HPO_4_, 8 mM NaCl, 1 mM Mg_2_SO_4_, and 0.1 mM CaCl_2_, supplemented with 0.1% Casamino Acids and 0.5% glucose and 0.5% agar. An aliquot of overnight bacterial cultures (~2.5 µL) was used to inoculate the center of each plate followed by incubation at 37°C for 24–48 h. The swarming radius was calculated by measuring the diameter from the point of application to the furthest points of the branched structures or the width of the spreading. For each plate, an average of at least four branched structures or four directions from the center of the swarming area was measured and averaged.

Twitching assays were performed as previously described (72). Briefly, PAO1 WT and the *hemO* allelic strains were freshly streaked onto PIA plates and grown overnight at 37°C. LB plates with or without a 5 µM mix of BVIXβ/δ or BVIXα were solidified with 1% agar (10 mL/plate) and stab inoculated using a sterile 10-µL pipette tip and placed in a humidified 37°C chamber. After 48-h growth, twitching zones were visualized by removing the agar from the plate and flooding the petri dish with 1% (wt/vol) crystal violet solution. The plates were then incubated for 5 min and washed with distilled water. The circular twitching radius twitching was measured from the point of application to the crystal violet edge. For all motility assays, the average diameter was calculated from an *n* of 6 biological replicates (plates) per strain and condition.

### Biofilm formation and measurement

The biofilm assay was performed using the crystal violet method as previously described (73) using the minimal biofilm eradication concentration (MBEC) assay physiology and genetics biofilm plates. Briefly, 96-well MBEC plates were inoculated with 200 µL of PAO1 WT or the *hemO* allelic strains following 3 h of growth in M9 media to induce an iron-depleted state. Cultures were then supplemented with either 5 µM heme, FeCl_3_, a mix of BVIXβ/δ, or BVIXα. The MBEC plates were incubated at 37°C for 48 h. Following incubation, the peg lid was removed and washed with 0.9% saline solution to remove non-adherent cells. The pegs were then incubated in 0.1% crystal violet (wt/vol) solution to stain the adherent bacterial cells. Following incubation for 10 min, the pegs were washed with distilled water and left to dry for 30–40 min. After drying, the pegs were destained in 200 µL of 30% acetic acid in a fresh 96-well plate for 30 min. The 96-well plate was read in a Biotek plate reader at 595 nm to quantify the crystal violet dye as a direct measure of biofilm formation. For each strain and condition, a minimum of three biological replicates and six technical replicates were performed.

### TEM

Bacterial strains were grown on PIA plates and imaged as previously described (74). Briefly, a drop of filtered water was placed at the edge of an overnight colony of PAO1 WT or the *hemO* allelic strains. A glow-discharged, formvar carbon-stabilized grid was floated upside down on the drop for 45 s. The grid was rinsed with a drop of filtered water and stained with 2% aqueous phosphotungstic acid for 15 s. Excess stain was wicked off the grid with filter paper, and the grids were allowed too fully air-dry. Samples were imaged with an FEI tecnai T12 (Thermo Fisher) transmission electron microscope at 80 KV with an AMT bottom-mount camera.

### Western blot analysis of PilA

PAO1 and the *hemO* allelic cells were harvested using a sterile loop following overnight growth (16 h) on M9 heme plates containing 5 µM heme. The resulting pellets were resuspended in 250 µL of Bugbuster (Novagen). Cells were incubated at room temperature on a rotating platform for 20 min and then spun down and collected in a fresh Eppendorf tube. Total protein concentrations were determined using the Bio-Rad RCDC assay. Samples of the cell lysate (10 µg of total protein) and Chameleon duo pre-stained protein ladder in 4× protein sample loading buffer (LI-COR) were run on a 12% SDS-PAGE. Proteins were transferred by electrophoresis to low-fluorescence polyvinylidene difluoride membrane (Bio-Rad) for Western blot analysis. Membranes were then stained with Revert 700 Total Protein Stain (LI-COR) to measure the total protein levels on the 700-nm channel of the OdysseyM (LI-COR) imaging system. Stained membranes were destained with Revert Destaining Solution (LI-COR) prior to imaging. The membranes were then blocked with blocking buffer (5% [wt/vol] skim milk in Tris-buffered saline (TBS) with 0.1% [vol/vol] Tween 20), washed, and probed with a 1:5,000 dilution of anti-PilA primary antibodies (75) in hybridization buffer (1%[wt/vol] skim milk in TBS with 0.2% [vol/vol] Tween 20). Membranes were rinsed three times in TBS with 0.2% (vol/vol) Tween 20 and probed with IRDye 800CW Goat Anti-Rabbit IgG (LI-COR) at a dilution of 1:25,000 in hybridization buffer. Proteins were visualized on the 800-nm channel of the OdysseyM (LI-COR) imaging system. Quantitation of PilA intensity normalized to total protein was performed using the Empiria Studio Software package. All experiments were performed on four biological replicates per strain and statistically analyzed by Student’s *t*-test.

### Quantitative label-free proteomics

PAO1 WT and *hemO* allelic strains were grown in shaking or static conditions as described above and harvested in late log phase at an OD_600_ of 1 (5 and 8 h for the WT and allelic strains, respectively). For static cultures, cells were removed from the plate with a sterile loop, and the total pellet weight was recorded in pre-weighed Eppendorf tubes. Sample preparation and quantitative label-free proteomics analyses were performed as previously described (76–79). In brief, cells were harvested by centrifugation at 2,000 rpm for 30 s at 4°C and then subsequently lysed in 4% sodium deoxycholate, reduced, alkylated, and trypsinolyzed on filter as previously described (80). Tryptic peptides were separated and analyzed on a nanoACQUITY ultra-performance liquid chromatography analytical column (BEH130 C_18_, 1.7 µm, 75 µm × 200 mm) (Waters Corporation, Milford, MA) over a 165-min linear acetonitrile gradient (3%–40%) with 0.1% formic acid using a nanoACQUITY UPLC system (Waters Corporation) coupled to an Orbitrap Fusion Lumos Tribrid mass spectrometer (Thermo Scientific, San Jose, CA). Full scans were acquired at a resolution of 240,000, and precursors were selected for fragmentation by higher-energy collisional dissociation (normalized collision energy at 35%) for a maximum 3-s cycle. Tandem mass spectra were searched against the *Pseudomonas* genome database PAO1 reference protein sequences (81) using the Sequest-HT and MS Amanda algorithms with a maximum precursor mass error tolerance of 10 ppm (82, 83). Carbamidomethylation of cysteine and deamidation of asparagine and glutamine were treated as static and dynamic modifications, respectively. Resulting hits were validated at a maximum false-discovery rate of 0.01 using the semi-supsemervised machine learning algorithm Percolator (83). Protein abundance ratios were measured by comparing the MS1 peak volumes of peptide ions whose identities were confirmed by MS2 sequencing. Label-free quantification was performed using an aligned accurate mass and retention time cluster quantification algorithm (Minora; Thermo Fisher Scientific, 2017). Gene function and pathway analysis was conducted using information from the *Pseudomonas* genome database (81), the *Pseudomonas* metabolome database (84), and the STRING database (28). Protein interactions were analyzed using STRING version 11.5 and visualized with Cytoscape version 3.8.0 (35).

### Quantitation of alkylquinolones, HSLs, and PHZs by LC-MS/MS

Samples from shaking culture (100 µL of supernatant) or static pellet 100 µL of 10 mg/mL were grown as described for the proteomic analysis and resuspended in PBS for extraction. Additionally, 5 µL of NHQ-d4 at 20 ng/mL in methanol was added as an internal standard to each sample. Samples were acidified with 200 µL of 1 N HCl and then extracted via liquid-liquid extraction using 700 µL methyl-tertbutyl ether (MtBE) and ethyl acetate, 1:1 (vol/vol). Samples were vortexed and then centrifuged at 3,000 rpm for 10 min. The top (organic) layer was transferred to a new set of tubes pre-spiked with 50 µL of dimethyl sulfoxide to mitigate adsorption as previously described (85). Samples were dried down at 30°C under nitrogen for approximately 20 min; DMSO will not evaporate under these conditions, leaving some residual liquid in the test tube. Samples were reconstituted with 200 µL of 5 mM picolinic acid with 0.1% formic acid in water:acetonitrile, (1:1. vol/vol) and transferred to low-volume autosampler inserts to reduce the surface area contacting the extracts.

Extracts were separated on an Ascentis Express C8 Column, 100 mm × 2.1 mm, 2.7 µm using an I-Class UPLC coupled to a TQ-XS triple quadrupole mass spectrometer (Waters Corporation). Analytes were separated using a gradient mobile phase, where mobile phase A was 5 mM picolinic acid in water with 0.1% formic acid, and mobile phase B was 0.1% formic acid in acetonitrile. Picolinic acid was used in the mobile phase as a buffer to help chelate metals inherent to the instrumentation system; PQS will lose peak shape integrity without a chelator in the mobile phase. At a flow of 0.5 mL/min, the starting composition of 10% B was held for 30 s and then ramped to 95% B over 7.5 min, held for 1 min, and then returned to 10% B for a total analytical run time of 10.5 min. Mass spectrometer parameters were as follows: capillary voltage, 3.50 kV; desolvation temperature, 650°C; desolvation gas flow, 900 L/h; cone gas flow, 150 L/h; and nebulizer, 7.0 bar. All analytes were detected in electrospray ionization+ mode. Cone and collision energy were optimized for each analyte. Mass transitions are as follows: 1-HP, precursor ion (*m*/*z*) 197.2, product ion (*m*/*z*) 169.1, cone 60 V, collision 25 eV; PCA, precursor ion (*m*/*z*) 225.0, product ion (*m*/*z*) 179.0, cone 20 V, collision 25 eV; 3-oxo-C8 HSL, precursor ion (*m*/*z*) 242.1, product ion (*m*/*z*) 141.0, cone 20 V, collision 15 eV; HHQ, precursor ion (*m*/*z*) 244.1, product ion (*m*/*z*) 159.1, cone 65 V, collision 35 eV; HQNO, precursor ion (*m*/*z*) 260.1, product ion (*m*/*z*) 159.1, cone 50 V, collision 30 eV; PQS-C7, precursor ion (*m*/*z*) 260.1, product ion (*m*/*z*) 175.1, cone 60 V, collision 23 eV; NHQ, precursor ion (*m*/*z*) 272.1, product ion (*m*/*z*) 159.1, cone 70 V, collision 35 eV; PQS-C9, precursor ion (*m*/*z*) 288.1, product ion (*m*/*z*) 175.1, cone 55 V, collision 30 eV; 3-oxo-C12 HSL, precursor ion (*m*/*z*) 298.1, product ion (*m*/*z*) 175.1, cone 55 V, collision 30 eV; C16-HSL, precursor ion (*m*/z) 244.1, product ion (*m*/*z*) 159.1, cone 65 V, collision 35 eV; and NHQ-d4 (IS), precursor ion (*m*/*z*) 276.3, product ion (*m*/*z*) 159.1, cone 65 V, collision 35 eV.

PYO quantitation was performed by absorbance spectroscopy as previously described (86). Shaking cultures of the PAO1 WT and *hemO* allelic strains were grown as described for the proteomics analysis. Aliquots (1–2 mL) were taken at an OD_600_ of 1 as for the proteomics experiments and pelleted. The supernatant was passed through a 0.2-µm filter, and the resulting solution was vortexed for 30 s to ensure oxidation of PYO prior to reading the absorbance at 691 nm (*A*_691_). The concentration of PYO was calculated from the absorbance using Beer’s law (*A* = ε*cl*, where *c* is concentration in millimolar, *l* is the path length in centimeters, and ε_mM_ is the millimolar extinction coefficient for oxidized PYO of 4.31 mM/cm).

### Quantitation of BVIXα, BVIXβ, and BVIXδ by LC-MS/MS

All strains were grown as described for the proteomics analysis. For shaken cultures, 1 mL of supernatant was taken for extraction at an OD_600_ of 1 as for the proteomics. For the shaken culture pellet and static pellet, pellets were resuspended to yield 1 mg/µL in PBS (i.e., 50 µL of resuspension solution was added to a 50-mg bacterial pellet), and then 25 µL of the resuspended pellet was combined with 975 µL of 200-µM butylated hydroxytoluene (BHT) in acetonitrile:water, 1:1 (vol/vol). BVIXα-d4 (Sigma) 10 µL of 50 ng/mL was added to each sample as an internal standard. The resulting solution was acidified with 100 µL of 1 N HCl followed by a liquid-liquid extraction using 1.5 mL of ethyl acetate. Samples were briefly vortexed and then centrifuged at 4°C for 10 min at 3,000 rpm. Using a dry-ice bath, the bottom (aqueous) layer was frozen, and the top (organic) layer was decanted into a new test tube. This step helped to minimize variable recovery due to emulsions. Samples were dried at ambient temperature under nitrogen gas (~25 min) and then reconstituted with 100 µL of 200 µM BHT in acetonitrile:water, 1:1 (vol/vol). We found that BHT is a necessary addition as an antioxidant to prevent non-specific oxidation of residual heme in samples since excreted metabolites can facilitate redox reactions.

Samples were analyzed using an I-Class UPLC coupled to a TQ-XS triple quadrupole mass spectrometer (Waters Corporation). An Acentis 90-Å RP amide column, 100 mm × 2.1 mm, 2.7 µm was used for chromatographic separation using a gradient separation with mobile phase A as 0.1% formic acid in water and mobile phase B as 0.1% formic acid in acetonitrile. The gradient separation started at 35% B and was held for 2 min, ramped to 45% B over 2 min, ramped to 95% B over 2.5 min, and then returned to 35% B for a total analytical run time of 10 min. Column switching was used such that the column eluent was only sent to the mass spectrometer from 2.5 to 5.5 min in the gradient program. Mass spectrometer parameters were as follows: capillary voltage, 2.50 kV; desolvation temperature, 550°C; desolvation gas flow, 900 L/h; cone gas flow, 150 L/h; and nebulizer, 7.0 bar. All analytes were detected in ESI + mode. Isomers were able to be distinguished based on unique fragmentation and retention time. Cone and collision energy were optimized for each analyte mass transition as follows: BVIXα, precursor ion (*m*/*z*) 583.4, product ion (*m*/*z*) 297.1, cone 35 V, collision 35 eV, and retention time 3.1 min; BVIXδ, precursor ion (*m*/*z*) 583.4, product ion (*m*/*z*) 343.0, cone 35 V, collision 35 eV, and retention time 3.3 min; BVIXβ, precursor ion (*m*/*z*) 583.4, product ion (*m*/*z*) 343.0, cone 35 V, collision 35 eV, and retention time 3.7 min; BVIXα-d4 (IS), precursor ion (*m*/*z*) 587.4, product ion (*m*/*z*) 299.1, cone 35 V, collision 35 eV, and retention time 3.1 min.

### Putative identification of rhamnolipids by UPLC-HDMS^E^

Static cell cultures were analyzed by resuspending pellets to yield 1 mg/µL in PBS. The resulting suspension was extracted using a modified Matyash extraction for total lipid extract. Briefly, 10 µL of EquiSPLASH (Avanti Polar Lipids) was added to 10 µL of pellet suspension to serve as internal standard. Ice-cold methanol (400 µL) was used to precipitate the proteins followed by an addition of 500 µL of ice-cold MtBE. Samples were incubated at 4°C for 6 min with vortexing at 650 rpm (ThermoMixer). At the end of the incubation, 500 µL of water was added and incubated with mixing for an additional 15 min. The samples were centrifuged at 8,000 × *g* for 8 min at 4°C. The organic (top) layer was transferred to a new test tube and 200 µL of MtBE was added to the aqueous (bottom) layer and allowed to mix while incubating for an additional 15 min. Samples were again centrifuged at 8,000 × *g* for 8 min at 4°C, and the top layer was combined with the top layer from the previous step. Samples were dried under nitrogen at 30°C (~20 min) and then resuspended in 100 µL acetonitrile:isopropanol:water (1:2:1) (vol/vol/vol).

Analysis was conducted according to the previously described methodology (87). Briefly, an ACQUITY H-Class UPLC equipped with a C18 CSH (2.1 × 100 mm, 1.7 µm) column was coupled to a Synapt G2S quadrupole-time-of-flight mass spectrometer equipped with traveling wave ion mobility (Waters Corporation). Rhamnolipids were separated using the following mobile phases: mobile phase A was 10-mM ammonium formate in water/acetonitrile (40:60, vol/vol) with 0.1% formic acid, and mobile phase B was 10-mM ammonium formate in acetonitrile/isopropanol (10:90, vol/vol) with 0.1% formic acid. Raw data were obtained for *m*/*z* of 100–1,800 for both positive and negative ionization modes. First scan was set at low collision energy and used to determine the precursor ion, while the second scan ramped from 30 to 55 eV in order to determine product ions. Leucine enkephalin (0.1 mg/mL in water-acetonitrile with 0.1% formic acid) was used to lock-mass to validate high mass accuracy data and infused at a flow rate of 7.5 µL/min. Raw data were imported into Progenesis QI (Nonlinear Dynamics), and species were putatively identified based on expected retention time and library match. MetLIN, NIST MS/MS, and LipidMAPS were all searched with a ±10-ppm tolerance for predicted precursor and fragmentation ions.

### ICP-MS analysis of intracellular iron concentrations

Aliquots (2 mL) were removed from cultures grown as described for the proteomics and metabolite analysis, and samples were removed at 2, 5, and 7 h, pelleted, and washed in fresh M9 media. The pellets were dissolved in trace metal-free ultrapure 20% HNO_3_ and boiled overnight at 100°C. Samples were further diluted with ultrapure water to a final concentration of 2% HNO_3_ and subjected to ICP-MS on an Agilent 7700 ICP-MS (Agilent Technologies). ICP-MS runs were calibrated with high-purity iron standard solution (Sigma-Aldrich), and raw ICP-MS data (ppb) were corrected for drift using values for scandium and germanium (CPI International) as internal standards and added to samples during processing. Corrected values were then normalized to culture density as determined by the absorbance at 600 nm. Experimental values and reported standard deviations were averages of *n* = 3–6 biological replicates.

## Data Availability

Proteomics data are deposited at ProteomeXchange via the PRIDE database with the project accession number PXD045835.

## SUPPLEMENTAL MATERIAL

Supplemental figures and tables mbio.02763-23-s0001.docx Figures S1 to S7 and Tables S2, S3, and S5 to S7.

Table S1 mbio.02763-23-s0002.xlsx Shaking proteomics data.

Table S4 mbio.02763-23-s0003.xlsx Static proteomics.

ASM does not own the copyrights to Supplemental Material that may be linked to, or accessed through, an article. The authors have granted ASM a non-exclusive, world-wide license to publish the Supplemental Material files. Please contact the corresponding author directly for reuse.
