# Supplementary material for: Pseudomonas aeruginosa heme metabolites biliverdin IXβ and IXδ are integral to lifestyle adaptations associated with chronic infection
Source: mBio. 2024 Feb 6;15(3):e02763-23. doi: 10.1128/mbio.02763-23 (PMC10936436; doi:10.1128/mbio.02763-23)
Supplement: Supplemental figures and tables — Figures S1 to S7 and Tables S2, S3, and S5 to S7. [file mbio.02763-23-s0001.docx]

**Supplementary Information**

*Pseudomonas aeruginosa* Heme Metabolites Biliverdin IXβ and IXδ are Integral to Lifestyle Adaptations Associated with Chronic Infection

Saba Shahzad, Samuel A. Krug, Susana Mouriño, Weiliang Huang, Maureen A. Kane* and Angela Wilks*

**Table S2.** Network analysis of significantly changed proteins in shaking conditions.

|  | Number of nodes^1^ | Number of edges^2^ | Expected number of edges^3^ | PPI  Enrichment  p-value |
| --- | --- | --- | --- | --- |
| *hemOα* upregulated | 193 | 331 | 107 | < 1 x 10^-16^ |
| *hemOα* downregulated | 96 | 91 | 35 | 3.22 x 10^-15^ |
| *hemOin* upregulated | 195 | 325 | 109 | < 1 x 10^-16^ |
| *hemOin* downregulated | 82 | 48 | 29 | 0.00104 |

**Table S3.** Network analysis of significantly changed proteins common to both *hemO* allelic strains in shaking and static conditions.

|  | Number of nodes^1^ | Number of edges^2^ | Expected number of edges^3^ | PPI  Enrichment  p-value |
| --- | --- | --- | --- | --- |
| ***hemO* strains shaking** upregulated | 145 | 228 | 56 | < 1 x 10^-16^ |
| downregulated  ***hemO* strains static** | 54 | 34 | 12 | 7.36 x 10^-8^ |
| upregulated | 128 | 214 | 86 | < 1 x 10^-16^ |
| downregulated | 203 | 552 | 159 | <1 x10^-16^ |
|  |  |  |  |  |

**Table S5.** Network analysis of significantly changed proteins in static conditions.

|  | Number of nodes^1^ | Number of edges^2^ | Expected number of edges^3^ | PPI  Enrichment  p-value |
| --- | --- | --- | --- | --- |
| *hemOα* upregulated | 207 | 326 | 184 | < 1 x 10^-16^ |
| *hemOα* downregulated | 312 | 771 | 346 | <1 x 10^-16^ |
| *hemOin* upregulated | 240 | 447 | 244 | < 1 x 10^-16^ |
| *hemOin* downregulated | 374 | 1060 | 470 | <1 x10^-16^ |
|  |  |  |  |  |

Table S6. Strains and plasmids used in this study.

| **Strains** | **Relevant genotype or description** | **Reference** |
| --- | --- | --- |

| ***E. coli*** | | | |
| --- | --- | --- | --- |
| S17-1-λ-pir  Nissle 1917 T7 (EcN(T7) | *pro thi hsdR*_ Tpr Smr; chromosome::RP4–2  Tc::Mu-Km::Tn7/λpir  EcN derivate, insertion of T7-RNA polymerase gene with *lac*UV5 promoter by deletion of *malEFG* operon | (1)  (2) |  |
| ***P. aeruginosa*** | | | |
| PAO1  PAO1 Δ*hemO*  PAO1 *hemO*α  PAO1 *hemO*in | Wild type  Chromosomal in frame *hemO* deletion in PAO1  Allelic exchange using PAO1 Δ*hemO*  Allelic exchange using PAO1 Δ*hemO* | (3)  (4)  (4)  (4) |  |
| **Plasmids** | | | |
| mini-CTX1-*hemO*α  mini-CTX1-*hemO*in  mini-CTX1-5’*hemO*α  mini-CTX1-5’*hemO*in  mini-CTX1-5’*hemO*α3’  mini-CTX1-5’*hemO*in3’  pEX18Tc  pEX18Tc-5’hemOα3’  pEX18Tc-5’*hemOin*3’  pE*hemO* | TcR; PCR-amplified *hemO*α coding sequence cloned as a EcoRI-HindIII fragment between same sites of mini-CTX1-P*hemO*  TcR; PCR-amplified *hemOin* coding sequence cloned as a EcoRI-HindIII fragment between same sites of mini-CTX1-P*hemO*  Tc^R^; allelic replacement vector  *P. aeruginosa hemO* gene cloned as NdeI-XhoI fragment into pET21a | (4)  (4)  This study  This study  This study  This study  (5)  This study  This study  (6) |  |

**Table S7.** Oligonucleotide primers and probes used in this study.

| **Name** | **Sequence** | **Reference** | |
| --- | --- | --- | --- |
| **RT-qPCR Primers and probes** | | |  |
| qPCR*-chpA* Probe  qPCR*-chpA* F qPCR*-chpA* R qPCR*-pilA* Probe  qPCR*-pilA* F qPCR*-pilA* R  qPCR*-pilJ* Probe  qPCR*-pilJ* F  qPCR*-pilJ* R qPCR-PA*16S* F  qPCR-PA*16S* R | 5'- /56-FAM/ACTGGCAGAGCCTGGATACCC T/3BHQ_1/-3'  5'- AACCTGCAACCGCTATATCC -3'  5'-AATACTCGACGCTGGTGATG -3'  5'- /56-FAM/TCGAGCCGGATCCAACAAGT T/3BHQ_1/-3'  5'- CTTCTCTGCGACCGAAACA -3'  5'- AATATCACCCGCACCACTATC -3'  5'- /56-FAM/TGATCCAGAACATCTCCAACGCCG /3BHQ_1/ -3'  5'-TGGAGGAGATCGAGAAGGTATC -3'  5'-GACGTTCATGGTGTTGGAAATG -3'  5'-GGTGGTTCAGCAAGTTGGATGTG-3'  5'-CCAGGTGGTCGCCTTCGC-3' | This study  This study  This Study  This study  This study  This study  This study  This study  This study  This study  This study | |
| **Site-directed mutagenesis** | | |  |
| *BamH*I-*hemO*-A  *EcoR*I-P*hemO*-R  *BseR*I-3’*hemO*  *Hind*III-3’*hemO*-R  HemO-F  HemO-R | 5’-GCGGATCCCGGGTTTCGATGCACAGGCG-3’  5’-GGGAATTCCTTTCGAGGGACGGAACGCA-3’  5’-AACGAGGAGGAAGAGCGCCTGGCCG-3’  5’-GCAAGCTTAGCAGCATGCCGAGACCGAG-3’  5’-GAATTCCCCTATGGATACCCTGGCCCC-3’  5’-AAAAAACCCCGGCAACCAGG-3’ | This study  This study  This study  This study  This study  This study | |

**Figure S1.** *Growth curves for PAO1 WT, hemOα and hemOin allelic strains.* A) in M9 supplemented with 5 μM heme*.* B) Growth curves as in A but grown in M9 (iron-deplete) or cultures supplemented with 5 μM FeCl_3_. Error bars represent the average of three biological replicates. Strains and conditions as shown in the figure legend.

**Figure S2.** *Biliverdin levels in the pellet and supernatants of PAO1 hemOα and hemOin allelic strains in shaking conditions.* LC-MS/MS quantification of the BVIX isomers when supplemented with 5 μM heme. Cells were harvested at OD600 of 1.0 as described for the proteomics experiments. BVIX values represent the standard deviation of *n*=6. *p* values as determined by Student *t* test comparing the BVIXβ and BVIXδ levels isomers between the pellets and supernatant within strain where *, *p* <0.05; **, and *p* <0.005.

**Figure S3.** *Swarming motility assays of PAO1 (A), hemOα (B), hemOin (C) strains in the absence or presence of the respective BVIX isomers.* Representative plates are shown from the *n*=6 used to generate the data in Fig 5. Assays were performed as described in Materials and Methods.

**Figure S4.** *Di-rhamnolipid* *identification and quantitation in PAO1 WT and hemO allelic strains.* A) Di-rhamnolipid core structure. B) LC-MS/MS quantitation of the major di-rhamnolipids in all strains. Extraction and analysis as described in Materials and Methods. Experiments show the standard deviation of *n* of 6. *p* values as determined by Student *t* test comparing the *hemO* allelic strains to PAO1 WT where*, *p* < 0.05; **, *p* <0.005; ***, *p*<0.001.

**Figure S5.** *Twitching motility assays of PAO1 (A), hemOα (B), hemOin (C) strains in the absence or presence of the respective BVIX isomers.* Representative plates are shown from the *n*=6 used to generate the data in Fig 5. Assays were performed as described in Materials and Methods.

**Figure S6.** *Relative biofilm formation as determined by crystal violet assay for the strains supplemented with either 5 μM heme or 5 μM FeCl_3_.* Biofilm assays were performed as described in the Materials and Methods on a minimum of *n*=3 for each strain. *p* values as determined by Student *t* test comparing the *hemO* allelic strains to PAO1 WT for each condition, where *, *p* <0.05; ****, p* <0.001; ****, *p*<0.0001.

**Figure S7.** *LC-MS/MS identification analysis of HSLs,* *AQs, and PHZs in static conditions.* A) Levels of 3-oxoC12-HSL in shaking and static conditions. B) Quantification of PQS quorum sensing molecules in static conditions. C) Quantification of phenazine metabolites in static conditions. Extraction and analysis as described in Materials and Methods. Experiments show the standard deviation of *n*=4 or n=6. *p* values as determined by Student *t* test comparing the *hemO* allelic strains to PAO1 WT where ns, not significant, **, *p* <0.005; ***, *p* <0.001; ****, *p* <0.0001.

***References***

1. V. de Lorenzo, K. N. Timmis, Analysis and construction of stable phenotypes in gram-negative bacteria with Tn5- and Tn10-derived minitransposons. *Methods Enzymol* **235**, 386-405 (1994).

2. K. Fiege, N. Frankenberg-Dinkel, Construction of a new T7 promoter compatible *Escherichia coli* Nissle 1917 strain for recombinant production of heme-dependent proteins. *Microb Cell Fact* **19**, 190 (2020).

3. B. W. Holloway, Genetic recombination in *Pseudomonas aeruginosa*. *J Gen Microbiol* **13**, 572-581 (1955).

4. S. Mourino, B. J. Giardina, H. Reyes-Caballero, A. Wilks, Metabolite-driven Regulation of Heme Uptake by the Biliverdin IXbeta/delta-Selective Heme Oxygenase (HemO) of *Pseudomonas aeruginosa*. *J Biol Chem* **291**, 20503-20515 (2016).

5. T. T. Hoang, R. R. Karkhoff-Schweizer, A. J. Kutchma, H. P. Schweizer, A broad-host-range Flp-FRT recombination system for site-specific excision of chromosomally-located DNA sequences: application for isolation of unmarked *Pseudomonas aeruginosa* mutants. *Gene* **212**, 77-86 (1998).

6. M. Ratliff, W. Zhu, R. Deshmukh, A. Wilks, I. Stojiljkovic, Homologues of neisserial heme oxygenase in gram-negative bacteria: degradation of heme by the product of the *pigA* gene of *Pseudomonas aeruginosa*. *J Bacteriol* **183**, 6394-6403 (2001).
